# Multidimensionally Programming Photoswitching via Orthogonal Covalent‐Noncovalent Chemistry for Dual‐Track Time‐Resolved Optical Encryption

**DOI:** 10.1002/advs.78337

**Published:** 2026-10-25

**Authors:** Qi Guo, Lewei Li, Meihui Yan, Yuzhao Yang, Dengchong Feng, Shaolin Lu, Cheng Wang, Min Cao, Haiqiang Wu, Zhongke Yuan, Dingshan Yu, Xudong Chen

**Affiliations:** ^1^ School of Chemical Engineering and Light Industry Guangdong University of Technology Guangzhou People's Republic of China; ^2^ Guangdong Laboratory of Chemistry and Chemical Engineering Jieyang Center Jieyang People's Republic of China; ^3^ Kingfa Science & Technology Co., Ltd. Guangzhou People's Republic of China; ^4^ School of Pharmacy Shenzhen University Medical School Shenzhen People's Republic of China; ^5^ Key Laboratory for Polymeric Composite and Functional Materials of Ministry of Education School of Chemistry Sun Yat‐sen University Guangzhou People's Republic of China

**Keywords:** donor–acceptor Stenhouse adducts, kinetic regulation, optical encryption, photochromic films, stimulus‐responsive materials

## Abstract

Encoding targeted information into the temporal evolution of optical states, rather than relying on static on/off states, is essential for storage, optical encryption and anti‐counterfeiting. However, achieving multiple independent information‐display processes remains a challenge. Herein, a “dual‐track” time‐resolved optical encryption strategy is realized based on multidimensional programming of donor–acceptor Stenhouse adduct (DASA) photoswitching through orthogonal covalent‐noncovalent chemistry. The temporal optical evolution during the photo‐bleaching and thermal‐recovery processes can be predictably programmed through the orthogonal combination of noncovalent interactions and covalent incorporation between DASAs and polyurethanes. Distinct from conventional strategies, noncovalent hydrogen‐bonding interactions synchronously suppress the photo‐bleaching rate (*k_photo_*) and thermal‐recovery rate (*k_dark_*) through isomerization confinement, whereas covalent incorporation reshapes the isomerization energetics, simultaneously accelerating *k_photo_* (2.4‐fold) and retarding *k_dark_* (60% decrease). This orthogonal regulation mode is generally applicable across diverse polyurethane chemistries and establishes a four‐quadrant kinetic map for DASA photoswitching, providing multiple encoding units with distinguishable temporal optical evolution. The photo‐bleaching and thermal‐recovery processes function as two independent information‐display tracks, allowing encrypted information to appear in either track, both tracks, or neither track. This work establishes a novel “dual‐track” paradigm for optical encryption by decoupling information in reversible photoswitching processes.

## Graphical abstract

A dual‐track time‐resolved encryption strategy with independent information display during photo‐bleaching and thermal‐recovery stages has been realized through multiple temporal encoding units derived from orthogonal covalent‐noncovalent programming of donor–acceptor Stenhouse adduct photoswitching. This strategy expands the information dimension of reversible stimuli‐responsive materials for advanced optical security applications.

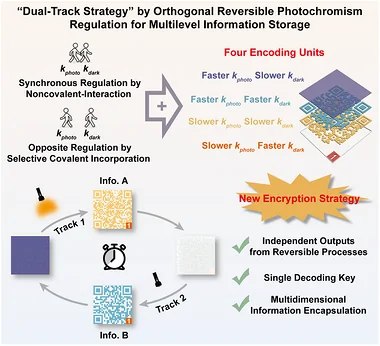

## 1 Introduction

The growing demand for anti‐counterfeiting and secure data storage has driven the development of optical encryption materials that go beyond traditional static‐switching [1, 2]. Time‐resolved optical encryption has emerged as a particularly promising strategy by introducing time as an additional encoding dimension to prevent unauthorized access. By exploiting differences in the response rates or color evolution of stimuli‐chromic [3, 4], fluorescence [5, 6], long‐afterglow [7, 8], and dynamic structural‐color materials [9, 10], encrypted information can be selectively displayed within specific temporal windows [11, 12]. However, most current approaches are fundamentally limited to a single dynamic information‐display process within each stimuli‐response cycle, thereby restricting further improvements in information capacity and security. Therefore, expanding the number of accessible information‐display processes is highly desirable for advancing time‐resolved optical encryption technology, yet remains a largely unmet challenge.

Molecular photoswitches, including azobenzene [13, 14], spiropyran [15, 16], diarylethene [17, 18], and donor–acceptor Stenhouse adducts (DASAs) [19, 20], are particularly attractive candidates for time‐resolved optical encryption, featuring non‐contact light activation, reversible switching, and pronounced optical contrast. Existing photoswitching‐based encryption strategies mainly rely on programming photoswitching rates to generate time‐resolved information displays [21, 22, 23, 24]. Although the reversible photoswitching processes of molecular photoswitches, in principle, offer the possibility of two independent information‐display processes, most strategies still rely on a single information‐display process. The recovery stage merely restores the initial optical state or reproduces information encoded during the photo‐responsive stage rather than generating independent information outputs. Although additional dynamic information displays can be introduced by integrating multiple materials, such approaches inevitably increase system complexity and require additional decoding stimuli [25, 26]. How to exploit the intrinsic reversibility of molecular photoswitches through programming photoswitching rates to achieve independent information display in the photo‐responsive and recovery stages therefore remains an open question.

Multidimensional programming of photoswitching rates through matrix engineering to generate encoding units with distinct temporal optical evolution behaviors represents a direct strategy toward decoupling the information of photo‐responsive and recovery stages [27]. DASAs provide an ideal platform for implementing this strategy because of their visible‐light‐induced photo‐bleaching, spontaneous thermal‐recovery, and exceptional responsiveness to the matrix environment [28]. In particular, the two reversible processes can be completed under a single visible‐light stimulus, thereby substantially simplifying the decoding procedure. Current matrix‐engineering strategies for DASA photoswitching mainly rely on tuning the free volume, noncovalent interactions, or steric confinement through either covalent incorporation into polymer chains [29], adjustment of polymer glass‐transition temperatures [30, 31] and block structures [32, 33], or encapsulation within porous frameworks [34, 35] and supramolecular hosts [36, 37]. Despite these advances, available regulation strategies generally cause the photo‐bleaching and thermal‐recovery rates to become simultaneously faster or slower. Consequently, one process cannot be selectively programmed while retaining the other, preventing independent dynamic information display in the photo‐bleaching and thermal‐recovery stages. Moreover, the lack of a quantitatively predictable regulation framework further hinders the rational design of DASA photoswitching. As such, it is pressingly required to figure out a feasible solution to establish a multidimensional structural‐performance relationship model, while understanding the effect of matrix environments on the photoswitching kinetics.

Herein, we propose an orthogonal covalent‐noncovalent strategy for multidimensional programming of DASA photoswitching to establish a novel “dual‐track” time‐resolved optical encryption paradigm. Two mechanistically distinct regulation modes are established between DASAs and segmented polyurethanes (PUs): noncovalent hydrogen‐bond engineering synchronously modulates both the photo‐bleaching rate (*k_photo_*) and the thermal‐recovery rate (*k_dark_*) by restricting the configurational and conformational rearrangements required for DASA isomerization, whereas covalent incorporation of DASA into the polymer backbone introduces a distinctive opposite regulation mode that simultaneously accelerates photo‐bleaching and retards thermal‐recovery by reshaping the intrinsic isomerization free‐energy landscape (Figure 1a,b). The coexistence of these two modes enables *k_photo_* and *k_dark_* to be multidimensionally programmed, as captured in a four‐quadrant kinetic map providing four temporally distinguishable encoding units (Figure 1c), denoted as FF (faster *k_photo_*/faster *k_dark_*), FS (faster *k_photo_*/slower *k_dark_*), SF (slower *k_photo_*/faster *k_dark_*), and SS (slower *k_photo_*/slower *k_dark_*). The resulting photochromic labels demonstrate independent dynamic information display in the photo‐bleaching and thermal recovery stages, enabling both planar and three‐dimensional dual‐track encryption without additional responsive components or decoding keys (Figure 1d). This work establishes a “dual‐track” time‐resolved encryption strategy that expands the information dimension accessible from reversible photoswitching processes for advanced optical security applications.

**FIGURE 1:**
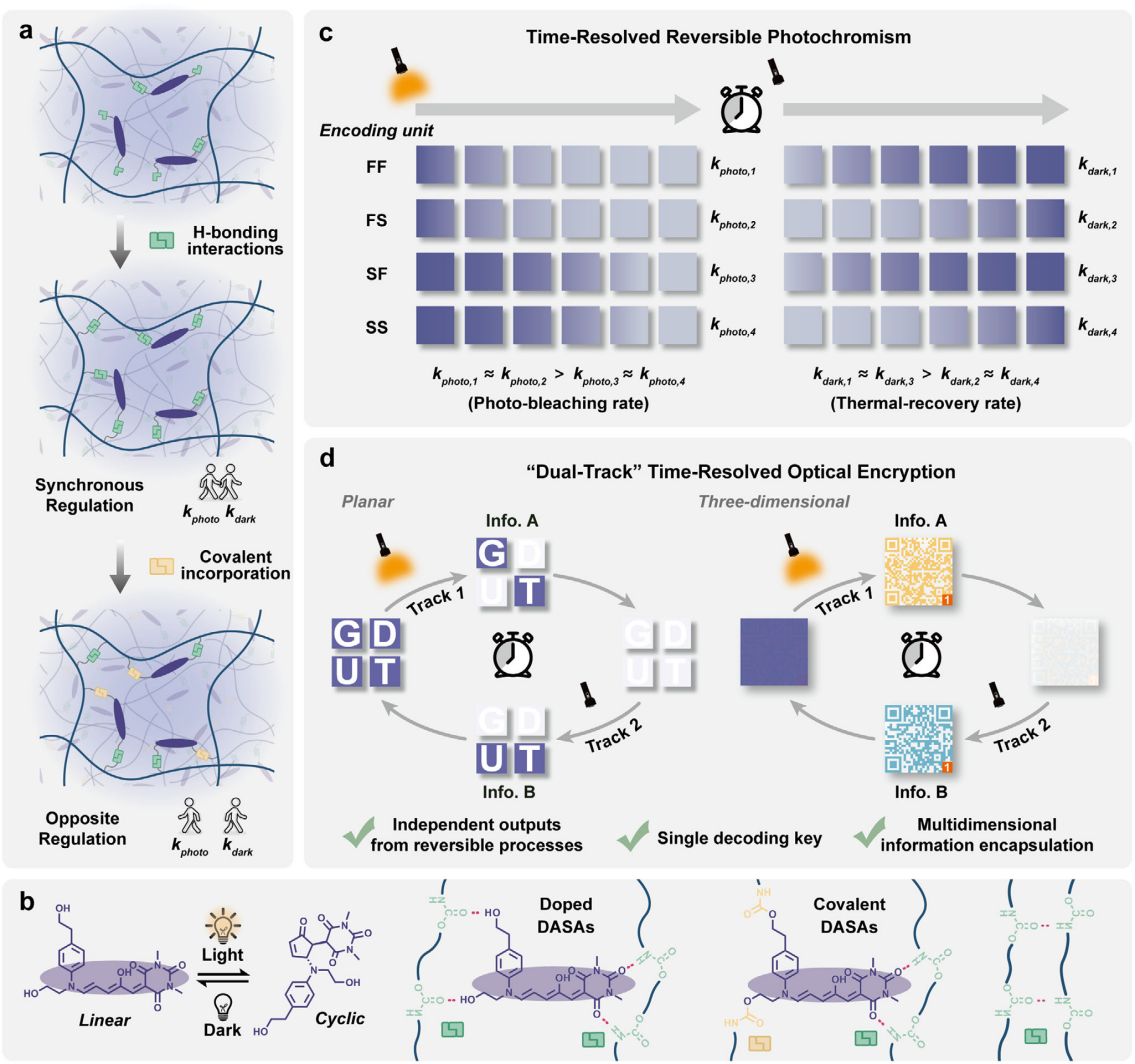
(a) Schematic illustration of orthogonal covalent‐noncovalent regulation in DASA‐2 photoswitching rates, comprising hydrogen‐bond‐mediated synchronous regulation and covalent‐incorporation‐induced opposite regulation. (b) Schematic illustration of reversible DASA‐2 photoswitching, its immobilization in polyurethane via hydrogen‐bonding interactions and covalent incorporation, and the intrinsic hydrogen‐bonding network of the PU matrix. (c) Time‐resolved reversible photochromism of the four encoding units. (d) Schematic illustration of the dual‐track time‐resolved optical encryption strategy implemented in both planar and three‐dimensional information architectures. In the three‐dimensional application schematic, different colors are used solely to distinguish the encoding units for clarity, while the actual photochromic films exhibit a similar blue‐purple appearance prior to irradiation, consistent with the planar schematic.

## 2 Results and Discussion

Achieving multidimensional programming of DASA photoswitching requires a material platform with tunable architectures and multiple coupled regulatory pathways. On this basis, the development of quantitative and predictive regulation models is crucial for enabling high‐dimensional optical encryption. To this end, a series of PUs with tunable chain structures were synthesized by two‐step polymerization using polytetramethylene ether glycol (PTMEG), dicyclohexylmethane‐4,4’‐diisocyanate (HMDI), and 1,4‐butanediol (1,4‐BDO), and are denoted as HMDI‐PTMEG*_x_*_:_ *_y_* (*x*:*y* = the molar ratio of PTMEG to 1,4‐BDO). A hydroxyl‐functionalized DASA derivative (DASA‐2) was synthesized through Knoevenagel condensation followed by amine methylation. The introduced hydroxyl groups provide additional noncovalent interaction sites with PUs in the physically doped state (denoted as DASA‐2@HMDI‐PTMEG*_x_*_:_ *_y_*), while also enabling DASA‐2 to be covalently incorporated into the polyurethane backbone through partial substitution of 1,4‐BDO during polymerization (denoted as DASA‐2‐HMDI‐PTMEG*_x_*_:_ *_y_*), providing two complementary regulation modes for DASA photoswitching.

The photophysical properties of physically doped DASA‐2@HMDI‐PTMEG_1:1_ and covalently incorporated DASA‐2‐HMDI‐PTMEG_1:1_ were first investigated by static and time‐dependent ultraviolet–visible (UV–Vis) absorption spectroscopy. As illustrated in Figure 2a, both materials exhibit nearly identical visible absorption bands centered at approximately 589 nm and typical thermally reversible (T‐type) negative photochromism, reversibly switching between the colored *linear* state and the colorless *cyclic* state under visible‐light irradiation and dark storage. Despite their similar initial optical properties, their photoswitching rates differ markedly. Upon white‐light irradiation (43 mW cm^−2^), the covalently incorporated system underwent substantially faster photo‐bleaching than its physically doped counterpart, reaching 50% conversion (*t*_1/2_) within only 10.3 s, compared with 28.4 s for DASA‐2@HMDI‐PTMEG_1:1_ at 35°C (Figure 2b). Conversely, thermal‐recovery was significantly retarded after covalent incorporation, with DASA‐2‐HMDI‐PTMEG_1:1_ requiring 287.4 s to recover 50% of its initial absorbance, whereas DASA‐2@HMDI‐PTMEG_1:1_ reached the same recovery level in only 95.5 s (Figure 2c).

**FIGURE 2:**
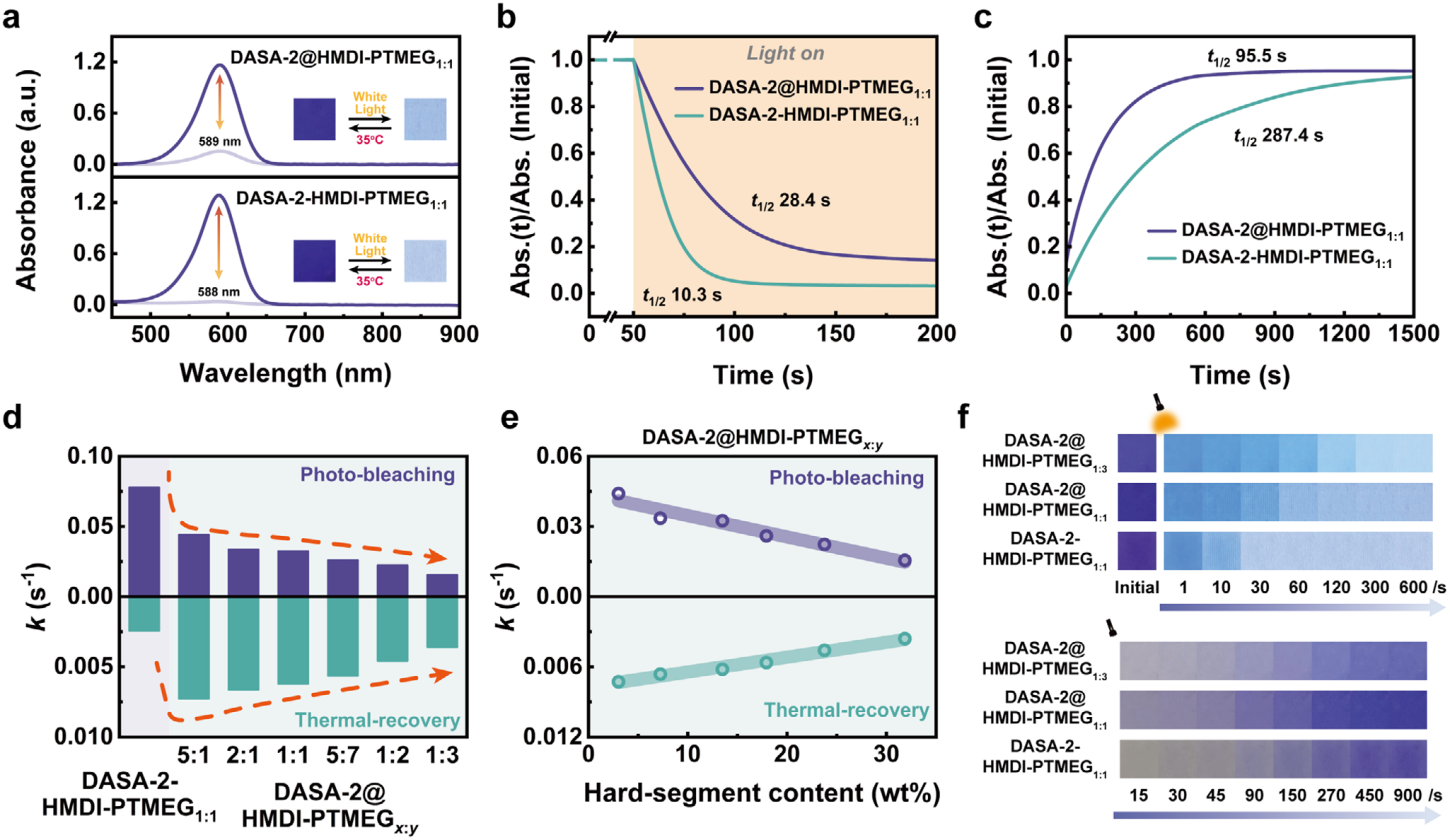
(a) UV–vis absorption spectra of DASA‐2 physically doped in HMDI‐PTMEG_1:1_ (top), and covalently incorporated into HMDI‐PTMEG_1:1_ (bottom). Time dependence evolution of the maximum absorption for DASA‐2@HMDI‐PTMEG_1:1_ and DASA‐2‐HMDI‐PTMEG_1:1_ during (b) photo‐bleaching under white‐light irradiation (43 mW cm^−2^) and (c) subsequent thermal‐recovery in the dark at 35°C. (d) Comparison of *k_photo_* (top) and *k_dark_* (bottom) of DASA‐2‐HMDI‐PTMEG_1:1_ and DASA‐2@HMDI‐PTMEG*_x_*_:_ *_y_* with different soft‐hard segment ratios. (e) Dependence of *k_photo_* (top, R^2^ > 0.94) and *k_dark_* (bottom, R^2^ > 0.95) on the hard‐segment content of DASA‐2@HMDI‐PTMEG*_x_*_:_ *_y_*. (f) Temporal color evolution of representative reversible photochromic films.

To quantitatively compare the photoswitching rates, the photo‐bleaching and thermal‐recovery processes were analyzed using a classical two‐state model (Equations S1 and S2) [38]. Considering absorption‐induced light attenuation, *k_photo_* values were compared at similar initial absorbance levels or obtained at equivalent initial absorbance through absorbance‐dependent fitting using our established methodology [39, 40]. Furthermore, our previous study showed that concentration‐induced aggregation did not appreciably contribute to the observed photo‐bleaching rate variation within the investigated range [39]. As summarized in Figure 2d, relative to the physically doped counterpart (Figure S10 and Table S5), covalent incorporation (Figure S11 and Table S6) increased *k_photo_* by 2.4‐fold (from 0.0325 to 0.0779 s^−^^1^), while decreasing *k_dark_* by nearly 60% (from 0.00621 to 0.00244 s^−^^1^). By comparison, varying the hard‐segment content in the physically doped DASA‐2@HMDI‐PTMEG*_x_*_:_ *_y_* series produced a distinctly different regulation trend (Figures S12−S16 and Tables S7−S11). Increasing the hard‐segment content (*x*:*y* from 5:1 to 1:3) synchronously decreased *k_photo_* and *k_dark_* from 0.0441 s^−1^ and 0.00724 s^−1^ to 0.0106 s^−1^ and 0.00424 s^−1^, respectively. To further quantify this trend, *k_photo_* and *k_dark_* were plotted as a function of the hard‐segment content for the physically doped DASA‐2@HMDI‐PTMEG*_x_*_:_ *_y_* series, and both exhibit excellent linear correlations (Figure 2e). Representative examples of both regulation modes are directly reflected in the temporal color evolution of the films, resulting in distinguishable time‐dependent color‐change behaviors (Figure 2f). Collectively, these results establish two distinct rate regulation modes: opposite modulation of photo‐bleaching and thermal‐recovery through selective covalent incorporation, and synchronous modulation of the two reversible processes through PU segmented‐structure engineering.

To understand the mechanism of the synchronous rate regulation observed in the physically doped DASA‐2@HMDI‐PTMEG*_x_*_:_ *_y_*, density functional theory (DFT) calculations together with attenuated total reflection‐Fourier transform infrared (ATR‐FTIR) spectroscopy were performed. Independent gradient model based on Hirshfeld partition (IGMH) and reduced density gradient (RDG) analyses [41, 42, 43, 44] reveal multiple hydrogen‐bonding interactions between DASA‐2 and the PU matrix in both the *linear* and *cyclic* states (Figure 3a and Figure S2). In particular, additional interactions are identified between carbamate groups and the key proton (H_d_) involved in thermal retro‐electrocyclization of the *cyclic* state. Such interactions could restrict the configurational and conformational rearrangements required for photoisomerization while stabilizing both the *linear* and *cyclic* states, thereby retarding photo‐bleaching and thermal‐recovery. The theoretical prediction was further verified experimentally by concentration‐dependent ATR‐FTIR spectra (Figure S3 and Table S1) combined with two‐dimensional (2D) correlation analysis [45, 46]. Because PUs inherently contain an extensive hydrogen‐bonding network, DASA‐2 concentration was employed as the perturbation variable to more directly resolve the reorganization of the PU hydrogen‐bonding states induced by DASA‐2. In the synchronous 2D correlation spectrum, the diagonal autopeaks at approximately 1719, 1692, and 1662 cm^−^^1^ were identified and assigned to free, disordered hydrogen‐bonded, and ordered hydrogen‐bonded carbonyl groups, respectively (Figure 3b). Based on the carbonyl deconvolution, the relative populations of these three components exhibit distinct variations with increasing DASA‐2 concentration, where the free and ordered hydrogen‐bonded carbonyl components decrease, whereas the disordered hydrogen‐bonded component increases. Correspondingly, the synchronous 2D correlation spectrum displays negative cross‐peaks at [1719, 1692 cm^−^^1^] and [1692, 1662 cm^−^^1^], indicating opposite changes between these carbonyl populations, together with a positive cross‐peak at [1719, 1662 cm^−^^1^], indicating synchronous variation. These correlated spectral changes demonstrate the redistribution of hydrogen‐bonding states in the PU matrix upon DASA‐2 incorporation. Furthermore, the positive cross peak at [1719, 1662 cm^−^^1^] in the asynchronous 2D correlation spectrum suggests the sequential response of these carbonyl species, indicating that DASA‐2 preferentially interacts with free carbonyl groups before perturbing the pre‐existing ordered hydrogen‐bonding network (Figure 3c).

**FIGURE 3:**
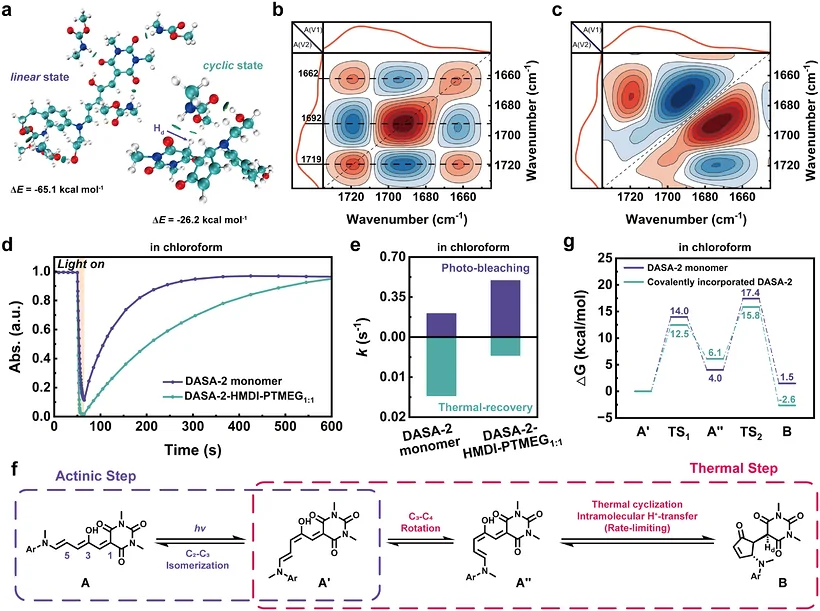
(a) IGMH maps (isovalue = 0.02) illustrating the interactions of *linear* and *cyclic* DASA‐2 with the carbamate moiety. (b) Synchronous and (c) asynchronous spectra of 2D ATR‐FTIR in the range of 1735–1645 cm^−1^. (d) Time‐resolved UV–vis absorption changes at the maximum absorption wavelength of DASA‐2 monomer and DASA‐2‐HMDI‐PTMEG_1:1_ in dilute chloroform solution during white‐light irradiation and subsequent dark recovery. (e) Comparison of *k_photo_* (top) and *k_dark_* (bottom) of DASA‐2 monomer and DASA‐2‐HMDI‐PTMEG_1:1_ in dilute chloroform solution. (f) Schematic illustration of the reversible photoisomerization pathway of DASAs. (g) Calculated free‐energy profiles of DASA‐2 monomer and covalently incorporated DASA‐2 during the thermal isomerization step.

To further validate the role of hydrogen bonding in synchronous rate regulation, two validation PUs (Valid‐O and Valid‐A) with systematically varied hydrogen‐bonding densities were designed through chain‐extender engineering (Figures S4, S17 and S18 and Tables S2, S12 and S13). In Valid‐O, replacing 1,4‐BDO with the sterically hindered 1,3‐BDO in HMDI‐PTMEG_1:1_ slightly weakened hydrogen‐bond formation (Figure S4a), leading to only minor increases in *k_photo_* (from 0.0325 to 0.0331 s^−1^) and *k_dark_* (from 0.00621 to 0.00641 s^−1^). In contrast, partial substitution of 1,4‐BDO with 1,4‐butanediamine (1,4‐BDA) in Valid‐A enhanced the hydrogen‐bond density through additional N–H groups (Figure S4b), which significantly suppressed both photo‐bleaching (*k_photo_*: 0.0112 s^−1^) and thermal‐recovery (*k_dark_*: 0.00573 s^−1^). The systematic dependence of the photoswitching rates on hydrogen‐bonding density establishes hydrogen bonding as the dominant mechanism underlying the synchronous regulation of DASA photoswitching behavior. This noncovalent regulation pathway provides a quantitatively predictable framework for synchronously programming photochromic rates through PU segmented‐structure engineering.

Unlike the hydrogen‐bond‐mediated synchronous regulation established above, covalent incorporation produces a fundamentally different photochromic behavior, accelerating photo‐bleaching while simultaneously retarding thermal‐recovery. To elucidate the origin of this opposite regulation mode, we first examined whether the observed rate changes arise from the solid‐state polymer matrix or from covalent incorporation itself. The intrinsic photoswitching rates of DASA‐2‐HMDI‐PTMEG_1:1_ and the DASA‐2 monomer were therefore compared in dilute chloroform solution, where solid‐state confinement and intermolecular interactions are largely eliminated. As shown in Figure 3d, DASA‐2‐HMDI‐PTMEG_1:1_ still exhibited markedly different photoswitching rates from DASA‐2 monomer, with *k_photo_* increasing from 0.204 to 0.493 s^−^^1^ and *k_dark_* decreasing from 0.0146 to 0.00455 s^−^^1^ (Figures 3e and S19 and Table S14). These results indicate that the opposite rate regulation induced by covalent incorporation cannot be solely attributed to differences in polymer microenvironments. Instead, covalent incorporation directly alters the intrinsic photoswitching energetics of DASA‐2.

To gain molecular‐level insight into this effect, DFT calculations were performed to compare the isomerization energy profiles of the DASA‐2 monomer and DASA‐2‐HMDI‐PTMEG_1:1_ in chloroform. For computational feasibility, a urethane‐functionalized DASA‐2 model was employed to represent the covalently incorporated DASA‐2 unit in DASA‐2‐HMDI‐PTMEG_1:1_. As shown in Figure 3f, the photo‐bleaching of typical DASAs involves photo‐induced double‐bond isomerization around C_2_‐C_3_ (A→A'), followed by σ‐bond rotation around C_3_‐C_4_ (A'→A'') and thermally driven 4π‐electrocyclization (A''→B), whereas thermal‐recovery proceeds through the reverse pathway. In the photoswitching pathway, the thermal step is generally the rate‐limiting process [47, 48]. The calculated energy profiles revealed that covalent incorporation lowered the activation barrier for the A′→A′′ isomerization step from 14.0 to 12.5 kcal mol^−1^ and for the A′′→B isomerization step from 13.4 to 9.7 kcal mol^−1^, facilitating the structural rearrangement required for cyclization and thereby contributing to the accelerated photo‐bleaching process (Figure 3g and Table S3). In contrast, the thermal‐recovery pathway becomes less favorable after covalent incorporation. For covalently incorporated DASA‐2, the free‐energy was decreased by 8.7 kcal mol^−1^ for A′′ to B step, larger than that for DASA‐2 monomer (2.5 kcal mol^−1^), indicating enhanced thermodynamic stabilization of the *cyclic* state. Meanwhile, the activation barrier of the retro‐electrocyclization step (B→A′′) increases from 15.9 to 18.4 kcal mol^−1^. The combined effects of a more stable cyclic state and a higher ring‐opening barrier therefore retard the thermal‐recovery process. These results demonstrate that covalent incorporation fundamentally reshapes the isomerization energetics of DASA photoswitching, thereby promoting photo‐bleaching while suppressing thermal‐recovery.

The distinct photoswitching regulation trends arising from noncovalent and covalent regulation together expand the accessible temporal evolution space of DASA reversible photochromism. To evaluate the generality of these two regulation modes and establish a comprehensive kinetic map of DASA photoswitching, additional PUs with different soft‐ and hard‐segment chemistries were systematically investigated (Figures S20–S35 and Tables S15–S30). Specifically, hydroxyl‐terminated poly(caprolactone) (PCL) and isophorone diisocyanate (IPDI) were introduced to replace the PTMEG soft‐segment and HMDI isocyanate, respectively, affording DASA‐2@HMDI‐PCL*_x_*_:_ *_y_*, DASA‐2@IPDI‐PTMEG*_x_*_:_ *_y_* and DASA‐2@IPDI‐PCL*_x_*_:_ *_y_*. As shown in Figure 4a–c, the synchronous decrease of *k_photo_* and *k_dark_* with increasing hard‐segment content was preserved in all PU series. Likewise, covalent incorporation of DASA‐2 into HMDI‐PCL_1:1_, IPDI‐PTMEG_1:1_ and IPDI‐PCL_1:1_ consistently induced faster *k_photo_* and slower *k_dark_* relative to the corresponding physically doped systems (Figure 4d). These results demonstrate that both the synchronous and opposite rate regulation strategies are broadly applicable across diverse PU chemistries. The coexistence of these two regulation modes suggests that *k_photo_* and *k_dark_* can be orthogonally programmed through the combination of hydrogen‐bonding engineering and selective covalent incorporation. Compared with previously reported DASA regulation strategies in polymer matrices, which generally produce only synchronous modulation of photo‐bleaching and thermal recovery or selectively regulate the photo‐bleaching process, the present approach expands the accessible rate regulation space of DASA photoswitching (Figure 4e).

**FIGURE 4:**
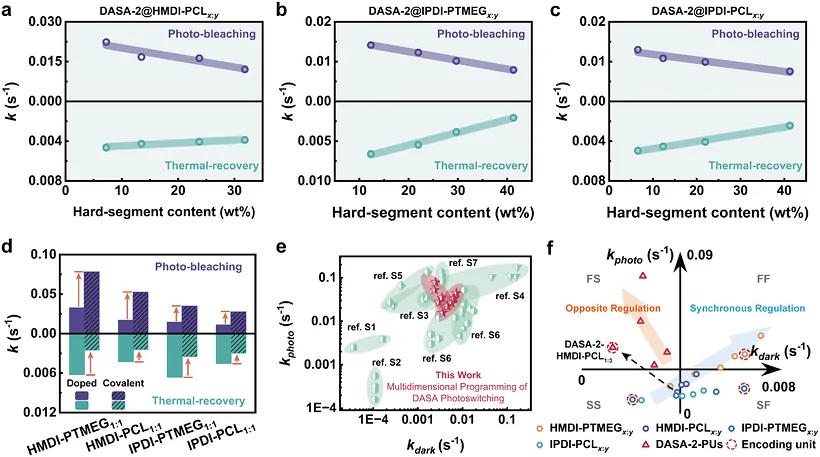
Dependence of *k_photo_* (top) and *k_dark_* (bottom) on hard‐segment content for (a) DASA‐2@HMDI‐PCL*_x_*_:_ *_y_*, (b) DASA‐2@IPDI‐PTMEG*_x_*_:_ *_y_* and (c) DASA‐2@IPDI‐PCL*_x_*_:_ *_y_*. (d) Effect of covalent incorporation on *k_photo_* (top) and *k_dark_* (bottom) of DASA‐2 in four representative PU matrices. (e) Photoswitching regulation comparison with previously reported DASAs in polymer matrices (Table S4). (f) Four‐quadrant kinetic map illustrating the synchronous and opposite regulation modes of DASA photoswitching.

Having established the generality of both regulation pathways, all investigated materials were projected onto a two‐dimensional kinetic map using *k_photo_* and *k_dark_* as coordinates (Figure 4f). To facilitate the selection of kinetically distinguishable encoding units for subsequent optical encryption, an operational classification was established under the fixed measurement conditions used in this work. Empirical thresholds of *k_photo_* = 0.025 s^−^^1^ and *k_dark_* = 0.004 s^−^^1^ were selected as intermediate values within the corresponding rate ranges of the initial material library, separating relatively fast and slow photo‐bleaching and thermal‐recovery behaviors. Accordingly, four kinetic regimes were defined: fast photo‐bleaching/fast thermal‐recovery (FF), fast photo‐bleaching/slow thermal‐recovery (FS), slow photo‐bleaching/fast thermal‐recovery (SF), and slow photo‐bleaching/slow thermal‐recovery (SS). This classification is intended as a practical framework for encoding rather than as an intrinsic boundary between different material states. Notably, hydrogen‐bond‐mediated regulation readily generated photochromic behavior distributed along the FF‐SS diagonal, spanning the intermediate SF region, whereas covalent incorporation shifted photochromic behavior toward the FS direction. Together, these two complementary regulation pathways provide practical access to distinct regions of the kinetic map.

To demonstrate the applicability of the DASA‐PU platform for optical information storage, the photo‐patterning capability of DASA photochromic films was first explored [49, 50]. Exploiting the inherent photodegradation of DASAs, both mask lithography and laser direct writing enabled high‐resolution patterning in the photochromic films (Figures 5a and S5). Both physically doped and covalently incorporated samples exhibited a pronounced decrease in their characteristic visible absorption bands after UV irradiation (Figure S6), indicating similar chromophore degradation behaviors. Based on our previous identification of amine‐donor‐derived derivatives as the predominant degradation products [49], both systems are expected to undergo similar degradation pathways involving disruption of the conjugated triene structure. Furthermore, gel permeation chromatography (GPC) analysis revealed no appreciable change in the molecular weight distribution of DASA‐2‐HMDI‐PTMEG_1:1_ after UV irradiation (Figure S7), suggesting that the polyurethane backbone remained largely intact. Resolution testing with a standard United States Air Force (USAF) resolution test 1951 target revealed a patterning resolution exceeding 8 line pairs per millimeter (Figure 5b), with further improvement anticipated through optimization of light collimation and mask precision. Based on this capability, a flexible quick response (QR) code label was fabricated for daily anti‐counterfeiting applications (Figure 5c). Upon exposure to a household flashlight, the label switched from a scannable to a concealed state, followed by complete spontaneous thermal‐recovery after 15 min at 35°C.

**FIGURE 5:**
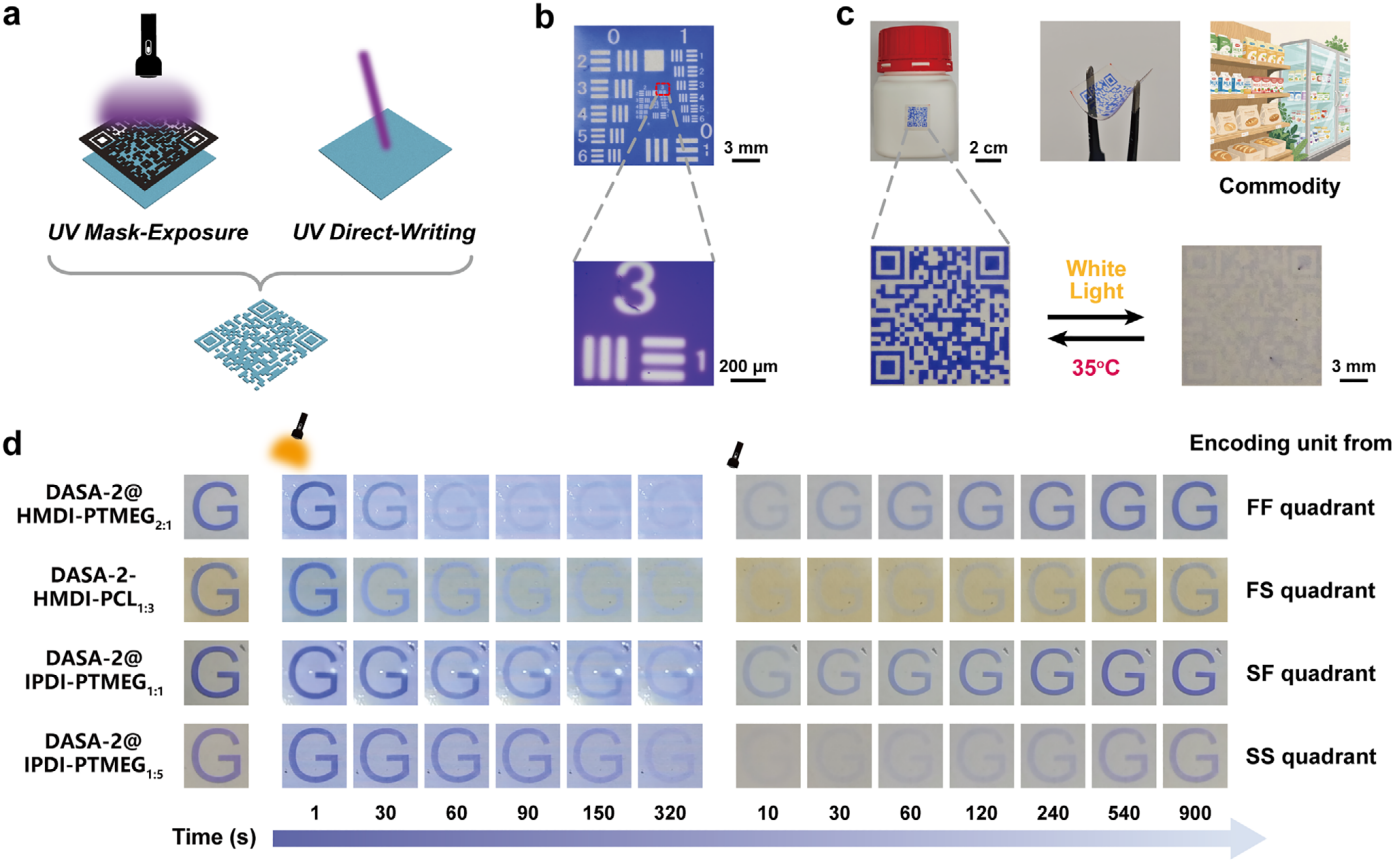
(a) Schematic illustration of permanent pattern generation based on the UV‐induced photodegradation of DASAs. (b) Resolution evaluation of the patterned film using the USAF 1951 Test Target Standard. (c) A flexible DASA‐2‐based QR code label integrated onto a commercial product, showing reversible photochromic response. (d) Time‐dependent color evolution of the four representative encoding units (FF, FS, SF, and SS) recorded in patterned films, highlighting their distinct reversible photochromic behavior for subsequent time‐resolved information encryption.

The kinetic map established above indicates that information can be selectively encoded by matching one rate while retaining differences in the other rate. In principle, four temporal encoding behaviors can be realized, corresponding to information appearing only during the photo‐bleaching stage, the thermal‐recovery stage, both stages, or neither stage. To achieve these encoding units, four kinetically distinguishable photochromic materials corresponding to the FF, FS, SF and SS regions of the kinetic map are required. Among the materials investigated here, FF, SF and SS units were readily accessible through hydrogen‐bond‐mediated synchronous regulation. However, a suitable material fulfilling the rate requirements of the FS unit was absent from the established material library. Guided by the orthogonal regulation principle, DASA‐2 was therefore covalently incorporated into HMDI‐PCL_1:3_ (Figure S43). As expected, the resulting DASA‐2‐HMDI‐PCL_1:3_ exhibited a pronounced shift in photoswitching rates, with *k_photo_* and *k_dark_* changing from 0.01206 s^−^^1^ and 0.00375 s^−^^1^ to 0.0374 s^−^^1^ and 0.00126 s^−^^1^, respectively, successfully generating the missing FS encoding unit (Figure 4f). Accordingly, DASA‐2@HMDI‐PTMEG_2:1_ from FF, DASA‐2‐HMDI‐PCL_1:3_ from FS, DASA‐2@IPDI‐PTMEG_1:1_ from SF, and DASA‐2@IPDI‐PTMEG_1:5_ from SS were selected as representative encoding units. Although these materials exhibit nearly identical initial appearances, they display markedly different temporal color‐evolution behaviors during reversible photochromism, allowing the photo‐bleaching and thermal‐recovery stages to be independently exploited as two information tracks (Figure 5d).

By assigning the four encoding units to different patterned regions, two time‐resolved optical labels displaying “GDUT” and “SYSU” were fabricated (Figure 6a). Prior to irradiation, all characters were simultaneously visible. Upon irradiation for approximately 30 s, rapid photo‐bleaching of the FF and FS units revealed the hidden information “GT” and “SU” within the photo‐bleaching information track. Continued irradiation gradually erased the remaining characters, leading to complete disappearance of the entire label after approximately 1 min. Subsequent thermal‐recovery at 30°C generated a second information track. After approximately 2 min in the dark, rapid thermal‐recovery of the FF and SF units revealed the hidden information “UT” and “YU”. Further thermal‐recovery restored all four encoding units, resulting in complete reconstruction of the original “GDUT” and “SYSU” patterns after approximately 20 min. These results demonstrate that independent information can be programmed into the photo‐bleaching and thermal‐recovery tracks, enabling “dual‐track” time‐resolved optical encryption with four temporal encoding units.

**FIGURE 6:**
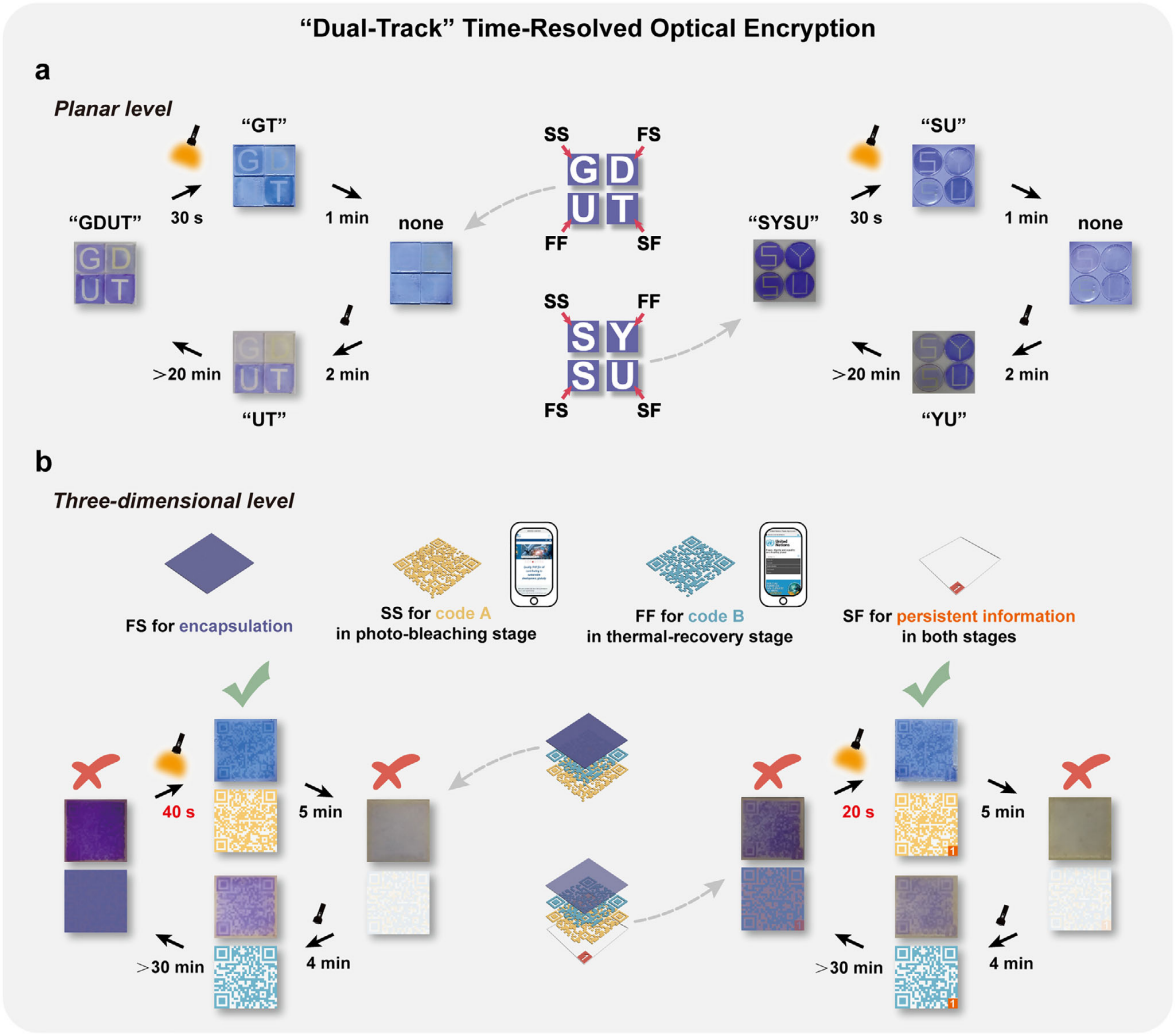
(a) Planar dual‐track optical encryption based on the four representative encoding units. Experimental photographs of patterned labels displaying “GDUT” (left) and “SYSU” (right) during reversible photochromism, together with the corresponding combinations of encoding units used for information display (middle). (b) Three‐dimensional dual‐track optical encryption enabled by layered information encapsulation. The left panels show a three‐layer architecture with a low‐absorbance encapsulation layer, whereas the right panels show a high‐absorbance encapsulation architecture integrated with a permanent information layer. Experimental photographs (top) and schematic illustrations (bottom) reveal the sequential appearance of distinct information during the photo‐bleaching and thermal‐recovery stages.

Beyond planar information display, the “dual‐track” strategy also enables temporal information stacking for multidimensional information storage. As illustrated in Figure 6b, the FS unit was employed as an encapsulation layer, the SS and FF units served as independently addressable information layers, and the SF unit functioned as a persistent information layer. Notably, the FS unit exhibited the lowest residual absorbance at the photostationary state (Equation S3) among all encoding units, which can be attributed to its unique combination of the highest *k_photo_* and the lowest *k_dark_* (Figure S8). Therefore, the FS unit can be almost completely erased under irradiation, thereby minimizing optical interference with the underlying information layers.

Based on this design, QR code A was encoded in the SS layer, while QR code B was encoded in the FF layer (Figures 6b and S9). Before irradiation, both QR codes remained concealed beneath the encapsulation layer and could not be independently decoded owing to mutual optical interference. Upon light exposure, the encapsulation layer together with QR code B was preferentially erased, revealing QR code A, which could be readily decoded using a smartphone scanner. Continued irradiation eventually erased all photochromic layers. During the subsequent thermal‐recovery stage at 30°C, QR code B reappeared preferentially owing to the faster thermal‐recovery rate of the FF layer and could likewise be independently decoded without interference from QR code A (Movie S1). The decoding time of photo‐bleaching stage could be further programmed by adjusting the absorption of the encapsulation layer. For example, decreasing the absorbance of the FS layer shortened the appearance time of QR code A from approximately 40 to 20 s. Meanwhile, information encoded in the SF unit remained visible throughout both information tracks and could either function as a persistent identifier or be integrated into the QR‐code architecture while maintaining reliable decoding performance. These results demonstrate that the orthogonal regulation of photochromic rates enables temporal information multiplexing and three‐dimensional information encapsulation, substantially enhancing both information capacity and security. More importantly, the multilayer information architecture is achieved without introducing additional responsive components or external stimuli, relying solely on programmable rate differences within a single reversible photochromic platform.

## 3 Conclusion

In summary, we have demonstrated a multidimensional programming strategy for DASA photoswitching through orthogonal covalent‐noncovalent chemistry between DASAs and segmented polyurethanes, enabling a novel “dual‐track” time‐resolved optical encryption paradigm with independent dynamic information display in the photo‐bleaching and thermal‐recovery stages. Noncovalent hydrogen‐bonding interactions synchronously modulate the *k_photo_* and *k_dark_* by restricting molecular isomerization, while segmented‐structure engineering of polyurethanes establishes a quantitatively predictable route for programming these rates. In contrast, covalent incorporation alters the intrinsic isomerization free‐energy landscape of DASA, resulting in an opposite regulation trend that can be orthogonally superimposed onto the noncovalent regulation mode. Their orthogonal combination enables multidimensional programming of DASA photoswitching, allowing the *k_photo_* and *k_dark_* to be independently matched and further providing arbitrary combinations of temporal optical evolution behaviors for information encoding. The generality of both regulation pathways across different PU systems enables the construction of a four‐quadrant kinetic map composed of photochromic units with differentiated temporal responses. Guided by this map, “dual‐track” time‐resolved optical encryption was realized at both planar and three‐dimensional levels by using four temporal encoding units. Moreover, the two completely independent information‐display processes can be decoded using only a single visible‐light decoding key. We anticipate that the concept of independent information display within reversible response processes will inspire new methodologies for expanding the information dimension of photoswitching and reversible stimuli‐responsive materials, providing opportunities for advanced optical information storage, anti‐counterfeiting technologies, dynamic information security systems, and beyond.

## 4 Experimental Section

The synthesis and the characterization of DASA‐2 and PU are detailed in the Supporting Information (Figures S32−S38 and Tables S31–S32). Procedures for films preparation and DFT calculations are given below.

### 4.1 Preparation of Photochromic Polymer Films

For DASAs@PU films, the as‐prepared dry PU materials were dissolved in tetrahydrofuran (10 wt.%), followed by the addition of DASAs (1 wt.%) to the polymer solution. The mixtures were vortexed for 30 min and sonicated for 10 min. Finally, the reversible photochromic solutions were drop‐coated onto horizontal quartz substrates (20 mm × 20 mm) and vacuum‐dried at 30°C for 24 h.

### 4.2 DFT Calculation

All calculations were performed with the Gaussian16 C.01 program by using a similar DFT calculation approach as in previous reports [10, 47, 48]. For calculating Gibbs free energy, the ground state (GS) structures of twisted intermediate species A' and A'', and *cyclic* form B were optimized by the M06‐2X method using the 6–31G(d) basis set with the 3rd generation Grimme dispersion correction (GD3). All minima were checked against the presence of imaginary frequencies. The GS transition states (TS) structures of TS_1_ (A'→A'') and TS_2_ (A''→B) were obtained at the same level. The solvent effects were included by using the universal continuum solvation model based on solute electron density (SMD). The higher precision electronic energy was calculated by using M06‐2X functional with ma‐def2‐TZVPP basis set. Furthermore, the Gibbs free energies of A', TS_1_, A'', TS_2_ and B forms were calculated by a Shermo program, combined with thermal energy correction and the scale factor of harmonic frequencies (ZPE) [51]. In polymer‐related calculations, Diethyl ether was utilized to represent PU soft segment, and methyl methylcarbamate was utilized to represent PU hard segment. For analyzing the interaction between PU polymer chains and DASA‐2, at least 200 dimer structures generated using Molclus [41] are adopted as initial structures for the semi‐empirical quantum mechanical optimization using MOPAC2016 at the PM6‐DH+ level. The geometry optimization of the most stable dimer with the lowest energy was conducted at the M06‐2X/6‐31G(d) level with a DFT‐D3 dispersion correction. Subsequently, the interaction energy combined with counterpoise correction was calculated by using M06‐2X functional with ma‐def2‐TZVPP basis set. The IGMH analyses [42] and the RDG analyses were performed using Multiwfn [43], followed by plotting the IGMH maps with an isovalue of 0.02 using Visual Molecular Dynamics (VMD) program [44] to intuitively exhibit the interaction of dimer. The isosurface is colored on a blue‐green‐red scale according to sign(*λ*_2_)*ρ*, ranging from −0.04 to 0.04 a.u.

## Author Contributions

**Qi Guo**: investigation, data curation, conceptualization, writing – original draft. **Lewei Li**: data curation, investigation. **Meihui Yan**: validation, methodology. **Yuzhao Yang**: writing – review and editing, supervision, funding acquisition. **Dengchong Feng**: methodology, visualization. **Shaolin Lu**: formal analysis, visualization. **Cheng Wang**: project administration, supervision. **Min Cao**: supervision, resources. **Haiqiang Wu**: supervision, resources. **Zhongke Yuan**: investigation, methodology. **Dingshan Yu**: conceptualization, supervision, writing – review and editing. **Xudong Chen**: funding acquisition, writing – review and editing, supervision, conceptualization.

## Conflicts of Interest

The authors declare no conflicts of interest.

## Supporting information




**Supporting File 1**: advs78337‐sup‐0001‐SuppMat.docx.

**Supporting File 2**: advs78337‐sup‐0002‐MovieS1.mp4.

## Data Availability

The data that supports the findings of this study are available in the supplementary material of this article.

## References

[1] Z. Zhang , X. Hou , and M. Li , “Bioinspired Real‐Time Dynamic Responsive Structural Colors,” Polymer Science & Technology 1, no. 9 (2025): 748–771, 10.1021/polymscitech.5c00037. PMC13052642 42257184

[2] Y. Shen , X. Le , Y. Wu , and T. Chen , “Stimulus‐Responsive Polymer Materials Toward Multi‐Mode and Multi‐Level Information Anti‐Counterfeiting: Recent Advances and Future Challenges,” Chemical Society Reviews 53, no. 2 (2024): 606–623, 10.1039/d3cs00753g. 38099593

[3] Y. Y. Dai , L. H. Cao , B. Zhou , and Y. Yang , “Time‐Resolved Photochromic Ionic Hydrogen‐Bonded Organic Frameworks for Multimodal Advanced Information Protection,” ACS Applied Materials & Interfaces 18, no. 11 (2026): 16788–16798, 10.1021/acsami.5c25547. 41814881

[4] Z. Y. Bai , X. Y. Xu , J. B. Fu , D. Zhao , Y. G. Wang , and H. R. Li , “Photo‐Responsive Room‐Temperature Phosphorescent Films Exhibiting Photochromism and Time‐Dependent Phosphorescent Colors for Multilevel Anti‐Counterfeiting,” Advanced Science 13, no. 24 (2026): 23121, 10.1002/advs.202523121. PMC13116334 41773074

[5] P. Das , T. Das , S. Koley , et al., “Time‐Encoded Information Encryption With pH Clock Guided Broad‐Spectrum Emission by Dynamic Assemblies,” Angewandte Chemie International Edition 64, no. 2 (2025): 202414239, 10.1002/anie.202414239. 39171779

[6] C. Yu , Z. Jiang , J. Han , et al., “Fe^3+^/Zr^4+^ Dual‐Responsive Distinct Yet Reversible Fluorescence of Polymeric Hydrogels for Rewritable Information Encryption With Time‐Dependent Security,” Advanced Functional Materials 36, no. 1 (2026): 11045, 10.1002/adfm.202511045.

[7] X. Ran , Y. Pan , L. Qiao , et al., “Broadband and Dynamic Afterglow Photoluminescent Carbon Dots for Time‐Resolved Optical Information Coding and Encryption,” Carbon 234 (2025): 119980, 10.1016/j.carbon.2024.119980.

[8] C. Li , F. Jiao , L. Dong , et al., “Time‐Division Multiplexing Physical Unclonable Functions Based on Multicolor Phosphorescent Carbon Dots,” Advanced Materials 37, no. 26 (2025): 2502522, 10.1002/adma.202502522. 40223363

[9] Y. F. Ji , T. F. Song , and H. F. Yu , “Assembly‐Induced Dynamic Structural Color in a Host‐Guest System for Time‐Dependent Anticounterfeiting and Double‐Lock Encryption,” Angewandte Chemie International Edition 63, no. 26 (2024): 202401208, 10.1002/anie.202401208. 38597254

[10] D. Feng , Q. Guo , Z. Huang , et al., “Viscoelasticity‐Controlled Relaxation in Wrinkling Surface for Multistage Time‐Resolved Optical Information Encryption,” Advanced Materials 36, no. 23 (2024): 2314201, 10.1002/adma.202314201. 38444232

[11] J. Zhou , D. Liu , L. Li , M. Qi , G. Yin , and T. Chen , “Responsive Organic Room‐Temperature Phosphorescence Materials for Spatial‐Time‐Resolved Anti‐Counterfeiting,” Chinese Chemical Letters 35, no. 11 (2024): 109929, 10.1016/j.cclet.2024.109929.

[12] A. Gao , F. Sun , Y. Duan , et al., “Programmable Encryption Based on Photochromism of Spiropyrans and Donor–Acceptor Stenhouse Adducts,” Advanced Functional Materials 34, no. 26 (2024): 2316457, 10.1002/adfm.202316457.

[13] T. Dang , Z.‐Y. Zhang , and T. Li , “Visible‐Light‐Activated Heteroaryl Azoswitches: Toward a More Colorful Future,” Journal of the American Chemical Society 146, no. 29 (2024): 19609–19620, 10.1021/jacs.4c03135. 38991225

[14] T. Dang , D. Dong , J. Zhang , Y. He , Z.‐Y. Zhang , and T. Li , “Thiazolylazopyrazoles as Nonsymmetric Bis‐Heteroaryl Azo Switches: High‐Yield Visible‐Light Photoisomerization and Increased Z ‐Isomer Stability by o ‐Carbonylation,” Angewandte Chemie International Edition 62, no. 24 (2023): 202301992, 10.1002/anie.202301992. 36861377

[15] J. Z. Wang , Y. Y. Zi , H. T. Cai , C. H. Wu , and Y. H. Yang , “Recent Advances in Spiropyran‐Based Solid‐State Photochromic Materials: From Molecular Design to Functional Applications,” Advanced Optical Materials 13, no. 33 (2025): 02370, 10.1002/adom.202502370.

[16] M. H. Chak , L. Wimberger , B. Richardson , et al., “Photoswitchable Merocyanine‐Amphiphiles With Programmable Self‐Assembly Times,” Chemistry – A European Journal 31, no. 54 (2025): 02399, 10.1002/chem.202502399. PMC12462240 40827559

[17] Q. Ai , K. J. Lan , L. Li , Z. G. Liu , and X. G. Hu , “Beyond Photochromism: Alternative Stimuli to Trigger Diarylethenes Switching,” Advanced Science 11, no. 48 (2024): 2410524, 10.1002/advs.202410524. PMC11672267 39488786

[18] I. Bala , J. T. Plank , B. Balamut , D. Henry , A. R. Lippert , and I. Aprahamian , “Multi‐Stage and Multi‐Colour Liquid Crystal Reflections Using a Chiral Triptycene Photoswitchable Dopant,” Nature Chemistry 16 (2024): 2084–2090, 10.1038/s41557-024-01648-0. 39367064

[19] J. R. Hemmer , Z. A. Page , K. D. Clark , et al., “Controlling Dark Equilibria and Enhancing Donor–Acceptor Stenhouse Adduct Photoswitching Properties through Carbon Acid Design,” Journal of the American Chemical Society 140, no. 33 (2018): 10425–10429, 10.1021/jacs.8b06067. 30074782

[20] M. Clerc , S. Sandlass , O. Rifaie‐Graham , et al., “Visible Light‐Responsive Materials: The (Photo)Chemistry and Applications of Donor–Acceptor Stenhouse Adducts in Polymer Science,” Chemical Society Reviews 52, no. 23 (2023): 8245–8294, 10.1039/d3cs00508a. PMC10680135 37905554

[21] Y. Xie , X. Zhao , H. Wang , et al., “Hydrogen Bond‐Associated Photofluorochromism for Time‐Resolved Information Encryption and Anti‐counterfeiting,” Angewandte Chemie International Edition 64, no. 2 (2025): 202414846, 10.1002/anie.202414846. 39174491

[22] Y. Ai , Y. Fei , Z. Shu , et al., “Dynamic Assembly‐Induced Time‐Resolved Optical Switches for Rewritable Advanced Information Encryption,” Advanced Optical Materials 11 (2023): 2301047, 10.1002/adom.202301047.

[23] L. Hu , Z. Wang , Y. Yang , et al., “Optical, Thermal and Mechanical Activation of Spiropyrans Enhanced by Hydrogen Bonding Interactions and its Application in Time–Resolved encryption,” Surfaces and Interfaces 46 (2024): 104116, 10.1016/j.surfin.2024.104116.

[24] X. Su , H. Xu , Q. Guo , et al., “Chain and Condensed State Structure‐Directed Fabrication of Dynamic Surface Gratings in Azopolymers for Invisible Patterns and Multi‐Modal Anti‐Counterfeiting,” Science China Chemistry 68 (2025): 5930–5941, 10.1007/s11426-025-2743-4.

[25] L. Hu , Y. Gao , Q. Cai , et al., “Cholesterol‐Substituted Spiropyran: Photochromism, Thermochromism, Mechanochromism and its Application in Time‐Resolved Information Encryption,” Journal of Colloid and Interface Science 665 (2024): 545–553, 10.1016/j.jcis.2024.03.129. 38547635

[26] J. Zhang , C. Song , S. Zhang , et al., “Time‐Dependent Information Encryption in Liquid Crystalline Polymer With Programmable Glass Transition Temperature,” Advanced Functional Materials 34, no. 28 (2024): 2400030, 10.1002/adfm.202400030.

[27] F. Sun and D. Wang , “Toward Real‐World Applications: Promoting Fast and Efficient Photoswitching in the Solid State,” Journal of Materials Chemistry C 10, no. 37 (2022): 13700–13716, 10.1039/d2tc01345b.

[28] S. Menon , “From Color to Control: The Emerging Power of Stenhouse Photoadducts in Adaptive Molecular Systems,” Chemistry – A European Journal 32, no. 24 (2026): 70961, 10.1002/chem.70961. 41954000

[29] M. Clerc , C. Tekin , S. Ulrich , et al., “Donor–Acceptor Stenhouse Adduct‐Polydimethylsiloxane‐Conjugates for Enhanced Photoswitching in Bulk Polymers,” Macromolecular Rapid Communications 43, no. 15 (2022): 2200120, 10.1002/marc.202200120. 35396766

[30] M. M. Sroda , J. Lee , Y. Kwon , et al., “Role of Material Composition in Photothermal Actuation of DASA‐Based Polymers,” ACS Applied Polymer Materials 4, no. 1 (2022): 141–149, 10.1021/acsapm.1c01108.

[31] R. McDonough , N. Rudgley , O. Majewski , M. V. Perkins , R. A. Evans , and D. A. Lewis , “Photochromic Performance of Donor‐Acceptor Stenhouse Adducts in Polymer Binders and Solution,” ChemPhotoChem 6, no. 9 (2022): 202200076, 10.1002/cptc.202200076.

[32] J. E. Yap , N. Mallo , D. S. Thomas , J. E. Beves , and M. H. Stenzel , “Comparing Photoswitching of Acrylate or Methacrylate Polymers Conjugated With Donor–Acceptor Stenhouse Adducts,” Polymer Chemistry 10, no. 47 (2019): 6515–6522, 10.1039/c9py01345h.

[33] S. Ulrich , J. R. Hemmer , Z. A. Page , et al., “Visible Light‐Responsive DASA‐Polymer Conjugates,” ACS Macro Letters 6, no. 7 (2017): 738–742, 10.1021/acsmacrolett.7b00350. 35650854

[34] F. Sun , X. Xiong , A. Gao , et al., “Fast Photochromism in Solid: Microenvironment in Metal‐Organic Frameworks Promotes the Isomerization of Donor‐Acceptor Stenhouse Adducts,” Chemical Engineering Journal 427 (2021): 132037, 10.1016/j.cej.2021.132037.

[35] Y. Duan , F. Sun , A. Gao , et al., “Visible‐Light‐Responsive Crystalline Solids by Equipping Covalent Organic Frameworks With Donor‐Acceptor Stenhouse Adducts,” Chinese Journal of Chemistry 41, no. 19 (2023): 2469–2475, 10.1002/cjoc.202300140.

[36] F. G. Blandon‐Cumbreras , M. Jurtik , A. Zavodna , et al., “Reversible Photoswitching of Donor‐Acceptor Stenhouse Adducts in Water,” Journal of the American Chemical Society 148, no. 1 (2026): 130–134, 10.1021/jacs.5c19813. PMC12814171 41427772

[37] R. Saha , A. Devaraj , S. Bhattacharyya , S. Das , E. Zangrando , and P. S. Mukherjee , “Unusual Behavior of Donor–Acceptor Stenhouse Adducts in Confined Space of a Water‐Soluble Pd II8 Molecular Vessel,” Journal of the American Chemical Society 141, no. 21 (2019): 8638–8645, 10.1021/jacs.9b03924. 31050889

[38] B. F. Lui , N. T. Tierce , F. Tong , et al., “Unusual Concentration Dependence of the Photoisomerization Reaction in Donor‐Acceptor Stenhouse Adducts,” Photochemical & Photobiological Sciences 18, no. 6 (2019): 1587–1595, 10.1039/c9pp00130a. 31089646

[39] Q. Guo , Z. Huang , D. Feng , et al., “Precisely Regulation of Photochromism via Absorption‐Induced Photoreaction Attenuation and Light Intensity Dependence for Time‐Resolved Asymmetric Encryption,” Chemical Engineering Journal 499 (2024): 156196, 10.1016/j.cej.2024.156196.

[40] N. K. Razdan , T. C. Lin , and A. Bhan , “Concepts Relevant for the Kinetic Analysis of Reversible Reaction Systems,” Chemical Reviews 123, no. 6 (2023): 2950–3006, 10.1021/acs.chemrev.2c00510. 36802557

[41] T. Lu , “Visualization Analysis of Covalent and Noncovalent Interactions in Real Space,” Angewandte Chemie International Edition 64, no. 29 (2025): 202504895, 10.1002/anie.202504895. 40323713

[42] T. Lu and Q. Chen , “Independent Gradient Model Based on Hirshfeld Partition: A New Method for Visual Study of Interactions in Chemical Systems,” Journal of Computational Chemistry 43, no. 8 (2022): 539–555, 10.1002/jcc.26812. 35108407

[43] T. Lu and F. Chen , “Multiwfn: A Multifunctional Wavefunction Analyzer,” Journal of Computational Chemistry 33, no. 5 (2012): 580–592, 10.1002/jcc.22885. 22162017

[44] W. Humphrey , A. Dalke , and K. Schulten , “VMD: Visual Molecular Dynamics,” Journal of Molecular Graphics 14, no. 1 (1996): 33–38, 10.1016/0263-7855(96)00018-5. 8744570

[45] C. Li , J. Liu , X. Qiu , X. Yang , X. Huang , and X. Zhang , “Photoswitchable and Reversible Fluorescent Eutectogels for Conformal Information Encryption,” Angewandte Chemie International Edition 62, no. 46 (2023): 202313971, 10.1002/anie.202313971. 37792427

[46] Y. W. Wen , M. Li , L. F. Fan , M. Z. Rong , and M. Q. Zhang , “Imparting Ultrahigh Strength to Polymers via a New Concept Strategy of Construction of up to Duodecuple Hydrogen Bonding among Macromolecular Chains,” Advanced Materials 36, no. 35 (2024): 2406574, 10.1002/adma.202406574. 38948960

[47] M. M. Lerch , S. J. Wezenberg , W. Szymanski , and B. L. Feringa , “Unraveling the Photoswitching Mechanism in Donor–Acceptor Stenhouse Adducts,” Journal of the American Chemical Society 138, no. 20 (2016): 6344–6347, 10.1021/jacs.6b01722. 27152878

[48] M. M. Lerch , M. Di Donato , A. D. Laurent , et al., “Solvent Effects on the Actinic Step of Donor–Acceptor Stenhouse Adduct Photoswitching,” Angewandte Chemie International Edition 57, no. 27 (2018): 8063–8068, 10.1002/anie.201803058. PMC6055754 29845699

[49] M. Yan , Y. Cao , L. Dong , et al., “Dual‐Mode Regulation of Thermochromism in Polymer‐Dispersed Cholesteric Liquid Crystals for Photoaddressing Temperature‐Resolved Encryption,” Advanced Optical Materials 13, no. 18 (2025): 2500391, 10.1002/adom.202500391.

[50] R. McDonough , N. Rudgley , O. Majewski , M. V. Perkins , R. A. Evans , and D. A. Lewis , “Fatigue of Donor‐Acceptor Stenhouse Adducts in Polymer Matrices and Solution,” ChemPhotoChem 7, no. 4 (2023): 202200247, 10.1002/cptc.202200247.

[51] T. Lu and Q. Chen , “Shermo: A General Code for Calculating Molecular Thermochemistry Properties,” Computational and Theoretical Chemistry 1200 (2021): 113249, 10.1016/j.comptc.2021.113249.

