# Supplementary material for: Multidimensionally Programming Photoswitching via Orthogonal Covalent‐Noncovalent Chemistry for Dual‐Track Time‐Resolved Optical Encryption
Source: Adv Sci (Weinh). 2026 Oct 25:e78337. Online ahead of print. doi: 10.1002/advs.78337 (PMC13696525; doi:10.1002/advs.78337)
Supplement: Supplementary file 1 [file ADVS-9999-e78337-s001.docx]

Supporting Information

Multidimensionally Programming Photoswitching via Orthogonal Covalent-Noncovalent Chemistry for Dual-Track Time-Resolved Optical Encryption

*Qi Guo,^a^ Lewei Li,^a^ Meihui Yan,^a^ Yuzhao Yang,*^,a,b^ Dengchong Feng,^a^ Shaolin Lu,^a,b^ Cheng Wang,^a,b^ Min Cao,^d^ Haiqiang Wu,^e^ Zhongke Yuan,*^,a,b^ Dingshan Yu,*^,c^ and Xudong Chen^a,b,c^*

*^a^* School of Chemical Engineering and Light Industry, Guangdong University of Technology, Guangzhou, 510006, P. R. China

*^b^* Guangdong Laboratory of Chemistry and Chemical Engineering Jieyang Center, Jieyang 515200, P. R. China

*^c^* Key Laboratory for Polymeric Composite and Functional Materials of Ministry of Education, School of Chemistry, Sun Yat-sen University, Guangzhou 510006, P. R. China

*^d^* Kingfa Science & Technology Co., Ltd., Guangzhou 510663, P. R. China

*^e^* School of Pharmacy, Shenzhen University Medical School, Shenzhen 518055, P. R. China

**1. Experimental Procedures**

**1.1** **Chemicals and materials**

All the chemicals and reagents were bought from commercial source and used without purification. Deionized water was produced by the ultrapure water system (Sartorius Arium mini). Chloroform-*d* (CDCl_3_) and acetonitrile-*d*_3_(ACN-*d*_3_) were purchased from Cambridge Isotope Laboratories, Inc. Dichloromethane (DCM), ethanol (EtOH), ethyl acetate (EA) and petroleum ether (PE) were acquired from General Reagent Co., Ltd. 2-(4-aminophenyl)ethanol was provided by Bide Pharmatech Co.,Ltd. Anhydrous tetrahydrofuran (THF), anhydrous N,N-dimethylformamide (DMF), isophorone diisocyanate (IPDI) and methylene-bis(4-cyclohexylisocyanate) (HMDI) were supplied by Energy Chemical. Dibutyltin dilaurate (DBTDL), 1,1,1,3,3,3-hexafluoro-2-propanol (HFIP) and 1,3-dimethylbarbituric acid were obtained from Shanghai Aladdin Biochemical Technology Co., Ltd. Diethyl ether (Et_2_O) was supplied by Guangzhou Chemical Reagent Factory. 2-Furaldehyde, 2-bromoethanol, potassium carbonate (K_2_CO_3_), triethylamine (Et_3_N), 1,4-butanediol (1,4-BDO), 1,3-butanediol (1,3-BDO), 1,4-butanediamine (1,4-BDA), hydroxyl-terminated poly(caprolactone) (PCL, $\bar{M}_{n}$ ≈ 2,000 Da) and polytetramethylene ether glycol (PTMEG, $\bar{M}_{n}$ ≈ 2,000 Da) were purchased from Shanghai Macklin Biochemical Co., Ltd.

**1.2 Characterization instruments and methods**

UV/Vis data were obtained on an Ocean Optics Maya2000 fiber spectrophotometer with a BiaoQi Optoelectronics DH-2000-BAL tungsten halogen light source. Reversible photoswitching kinetics were measured using a time-resolved UV-Vis spectroscopic setup based on the simplified pump-probe configuration established in our previous work.[1] A high-power white LED (Yinnuo K2-P70, 6500 K) equipped with a collimating lens was used as the excitation source (see Section 2.2 and Figure S2c of Ref. [1] for its wavelength-dependent spectral irradiance distribution and calibration procedure). The NMR spectra were measured with Bruker Advance III HD 400 spectrometer and Bruker AVANCE III HD 600 spectrometer. The chemical shifts were relative to the residual solvent signals (CDCl_3_: *δ*_H_ 7.26 and *δ*_C_ 77.16; ACN-*d*_3_: *δ*_H_ 1.94 and *δ*_C_ 118.26). HR-ESI-MS data were collected on a Thermo Fisher LTQ Orbitrap Elite high-resolution mass spectrometer. Differential scanning calorimetry (DSC) measurements were carried out using a Netzsch DSC 214 polyma instrument. IR spectra were recorded on a Perkin Elmer Frontier FTIR spectrophotometer equipped with an attenuated total reflection (ATR) accessory. Molecular weights of the polymers were measured by a Breeze gel permeation chromatography (GPC) using DMF or THF as eluent and polystyrene standard for calibration.

**1.3 Synthesis of DASAs**[2]

1.3.1 Synthesis of 5-(furan-2-ylmethylene)-1,3-dimethylpyrimidine-2,4,6(1H,3H,5H)-trione (**S1**)

The synthesis route and spectral data of **S1** were detailed in our previous work.[1]

1.3.2 Synthesis of 2-(4-((2-hydroxyethyl)amino)phenyl)ethan-1-ol (**S2**)

2-(4-aminophenyl)ethanol (685.9 mg, 5.0 mmol, 1.0 eq.), 2-bromoethanol (624.8 mg, 5.0 mmol, 1.0 eq.) and K_2_CO_3_ (829.2 mg, 6.0 mmol, 1.2 eq.) were added to 15 mL EtOH and stirred at 50℃ for 36 h. The filtrate was collected by vacuum filtration. After removing the solvent under reduced pressure, the crude product was subjected to a silica gel column chromatography eluted with EA-PE (60:40, v/v) to obtain a colorless crystalline compound **S2** (243 mg, 27% yield). ^1^H NMR (400 MHz, CDCl_3_, *δ*/ppm): 7.03 (d, *J* = 8.4 Hz, 2H), 6.61 (d, *J* = 8.4 Hz, 2H), 3.79 (t, *J* = 5.2 Hz, 2H), 3.78 (t, *J* = 6.4 Hz, 2H), 3.26 (t, *J* = 5.2 Hz, 2H), 2.75 (t, *J* = 6.4 Hz, 2H); ^13^C NMR (100 MHz, CDCl_3_, *δ*/ppm): 146.9, 130.0, 127.7, 113.7, 64.0, 61.3, 46.4, 38.3.

1.3.3 Synthesis of **DASA-2**

**S1** (702.6 mg, 3.0 mmol, 1.0 eq.) and **S2** (543.7 mg, 3.0 mmol, 1.0 eq.) were dissolved in 6 mL HFIP/DCM (1:4, v/v) and stirred for 3 h. After removing the solvent under reduced pressure, the crude product was redissolved in THF (6 mL) and then added to vigorously stirring PE. The pure product was filtered and washed with Et_2_O (3 × 10 mL) and PE (3 × 10 mL) to afford a purple solid (258 mg, 20% yield). NMR characterization is of the *cyclic* **DASA-2**. ^1^H NMR (600 MHz, acetonitrile-*d*_3_, *δ*/ppm): 7.79 (dd, *J* = 6.0, 1.8 Hz, 1H), 7.03 (d, *J* = 9.0 Hz, 2H), 6.74 (d, *J* = 9.0 Hz, 2H), 6.38 (dd, *J* = 6.0, 2.4 Hz, 1H), 5.19 (m, 1H), 4.08 (d, *J* = 1.8 Hz, 1H), 3.65 (m, 2H), 3.54 (m, 2H), 3.40 (dd, *J* = 4.2, 1.8 Hz, 1H), 3.25, (m, 1H), 3.17 (s, 3H), 3.02 (m, 1H), 2.91 (s, 3H), 2.64 (t, *J* = 6.6 Hz, 2H); ^13^C NMR (150 MHz, acetonitrile-*d*_3_, *δ*/ppm): 203.9, 168.5, 167.9, 165.1, 151.7, 146.9, 139.9, 134.5, 130.6, 116.4, 66.0, 63.8, 59.6, 51.2, 48.6, 47.7, 38.9, 28.9, 28.8. HR-(-)ESI-MS: *m/z* 414.16726 [M - H]^-^ (calcd. for C_21_H_24_O_6_N_3_).

**1.4 Synthesis of polyurethanes (PUs)**

PTMEG (2.0 g, 1.0 mmol, 1.0 eq.) and a magnetic stirrer in a dried glass vessel was dried at 110℃ *in vacuo* for 2 h to remove any moisture. After being cooled to 70℃ in an oil bath, 1.1 mL DMF was added in PTMEG with HMDI (550.9 mg, 2.1 mmol, 2.1 eq.) and DBTDL (11 mg, 0.20wt%), and stirred for 2 h under a N_2_ atmosphere to obtain prepolymer. The prepolymer was further diluted with 3.0 mL DMF, and cooled to room temperature. 1,4-BDO (90.1 mg, 1.0 mmol, 1.0 eq.) as chain extender dissolved in DMF (2.3 mL) was added to the prepolymer, while the reaction was heated to 60℃. After chain extension reaction for 4 h, most of the TPU solution was dried at 80℃ for 24 h and placed at 80℃ *in vacuo* for 72 h to remove any solution. Eventually, the target **HMDI-PTMEG_1:1_** was produced.

For adjusting the hydrogen bonding density of **HMDI-PTMEG*_x_*_:_*_y_***, the ratio of the soft to hard segment (*x*:*y*) was changed to 5:1, 2:1, 5:7, 1:2 and 1:3, to prepare HMDI-PTMEG_5:1_, HMDI-PTMEG_2:1_, HMDI-PTMEG_5:7_, HMDI-PTMEG_1:2_ and HMDI-PTMEG_1:3_.

The synthesis route of the validation PUs for experimentally proving the influence of hydrogen bonds to DASAs reversible photochromism is similar to that of **HMDI-PTMEG_1:1_** (2.1 mmol HMDI, 1.0 mmol PTMEG and 1.0 mmol 1,4-BDO). In **Valid-O**, chain extender 1,4-BDO (90.1 mg, 1.0 mmol, 1.0 eq.) was replaced by equimolar 1,3-BDO (90.1 mg, 1.0 mmol, 1.0 eq), to raising the hindrance around hydrogen bonds. In **Valid-A**, 1,4-BDA (22.0 mg, 0.25 mmol, 0.25 eq.) and 1,4-BDO (67.6 mg, 0.75 mmol, 0.75 eq.) as the mixed chain extenders were utilized to product Valid-A with higher hydrogen bonding density. Due to the higher reactivity of 1,4-BDA, the temperature of chain extension step was adjustment to 40℃ for 1 h and then 60℃ for 4 h.

The additional PUs were obtained by using PCL ($\bar{M}_{n}$ ≈ 2000 Da) as the soft segment or IPDI as the diisocyanate for highlighting the universality of hydrogen bonding regulation strategy. Specifically, HMDI (550.9 mg, 2.1 mmol, 1.0 eq.) and PTMEG (2.0 g, 1.0 mmol, 1.0 eq.) in **HMDI-PTMEG*_x_*_:_*_y_*** were selectively replaced by equimolar IPDI (466.9 mg, 2.1 mmol, 1.0 eq.) and PCL (2.0 g, 1.0 mmol, 1.0 eq.) for **HMDI-PCL*_x_*_:_*_y_***, **IPDI-PTMEG*_x_*_:_*_y_*** and **IPDI-PCL*_x_*_:_*_y_***.

DASA-covalently incorporated polyurethanes (**DASA-2-HMDI-PTMEG*_x_*_:_*_y_*, DASA-2-HMDI-PCL*_x_*_:_*_y_***, **DASA-2-IPDI-PTMEG*_x_*_:_*_y_*** and **DASA-2-IPDI-PCL*_x_*_:_*_y_***) were synthesized using a modified PU polymerization procedure. DASA-2 (1.0 wt% relative to the polymer) was first reacted with the entire amount of diisocyanate (HMDI or IPDI) required for the corresponding formulation in THF in the presence of DBTDL at 55 °C for 1 h. At the beginning of this step, DASA-2 was only partially soluble in THF because of its limited solubility. As the reaction proceeded, the mixture gradually became homogeneous, indicating the formation of more soluble isocyanate-functionalized DASA intermediates. The entire reaction mixture was subsequently added to the soft-segment diol (PTMEG or PCL) and allowed to prepolymerize for 2 h. In the final chain-extension step, DASA-2 was treated as a diol component and therefore partially replaced the molar amount of 1,4-BDO used in the corresponding pristine PU formulation. The amount of 1,4-BDO was reduced accordingly to maintain the overall NCO/OH stoichiometric balance. The remaining 1,4-BDO was then added as the chain extender, and the reaction was continued for another 4 h to afford the DASA-covalently incorporated polyurethane.

**1.5 Preparation of Reversible Photochromic Polymer Films**

The physically doped DASA-2@PU films were prepared by solution doping and drop coating. Specifically, the as-prepared PUs were dissolved in THF to obtain 10 wt% polymer solutions, followed by the addition of DASA-2 at concentrations of 2–15 mg g^-1^. The mixtures were shaken for 30 min and sonicated for 10 min to obtain homogeneous photochromic solutions. The solutions were subsequently drop-coated onto horizontal quartz substrates and vacuum-dried at 30 ℃ for 24 h. The resulting films had a thickness of approximately 50 μm.

For the covalently incorporated DASA-2-PU films, the as-synthesized photochromic PU solutions (15 wt% in THF) were directly drop-coated onto horizontal quartz substrates and vacuum-dried under the same conditions (30 ℃ for 24 h). The film thickness was controlled by adjusting the volume of solution deposited and ranged from approximately 50 to 150 μm.

**1.6 Photopatterning of DASA Reversible Photochromic Films**

**Mask lithography.** Photomasks consisting of quartz substrates with patterned chromium light-blocking layers were used. Each photomask was placed in close contact with the DASA reversible photochromic film. The film was exposed to a 365 nm UV light source (LEDM-PM6HT-89AD) at an irradiance of 26 mW cm^-2^ for 6–10 min at room temperature.

**Laser direct writing.** The DASA reversible photochromic films were patterned using a 365 nm laser marking machine at room temperature. The laser power was set to 20% of its rated power (5 W), with a scanning speed of 20–40 mm s^-1^ and a pulse repetition frequency of 70 kHz.

**1.7 Kinetic calculations of two-state model**[1, 3-4]

The reversible photochromic process was analyzed using a classical two-state model by monitoring the time-dependent evolution of the visible absorbance under irradiation and during subsequent thermal recovery.[1] The model assumes a dynamic equilibrium between the *linear* and *cyclic* forms of DASA, where the interconversion follows first-order kinetics.

Under white-light irradiation, the rate equation describing the evolution of the *linear* state can be expressed as:

$$\frac{dN_{linear}}{dt}=-k_{1}N_{linear}+k_{2}N_{cyclic}$$

where $k_{1}$and $k_{2}$represent the forward (*linear*-to-*cyclic*) and reverse (*cyclic*-to-*linear*) rate constants, respectively.

Considering the conservation relationship,

$$N_{linear}+N_{cyclic}=N_{0}$$

and assuming that the visible absorbance is proportional to the population of the *linear* form, the time-dependent absorbance during the photo-bleaching process can be described by:

$$\begin{aligned} Abs.\left( t \right)\propto N_{linear}\left( t \right)=\frac{k_{dark}}{k_{photo}}N_{0}+\frac{xk_{photo}-k_{dark}}{k_{photo}}N_{0}e^{-k_{photo}*t}\#Eq. S1 \end{aligned}$$

where $N_{0}$ is the total DASA concentration, $x$ is the fraction of the *linear* form under the initial dark equilibrium, $k_{photo}=k_{1}+k_{2}$, and $k_{dark}=k_{2}$.

For the thermal-recovery process, the *linear*-to-*cyclic* isomerization becomes negligible in the absence of irradiation ($k_{1}\approx0$). Under this condition, the *cyclic*-state population decays according to first-order kinetics.

Since the absorbance of the *cyclic* form can be expressed as the difference between the recovered absorbance and the instantaneous absorbance, the thermal-recovery process can be fitted using:

$$\begin{aligned} Abs.\left( t \right)=Abs.\left( \mathrm{rec} \right)-\left( Abs.\left( \mathrm{rec} \right)-Abs.\left( \mathrm{PSS} \right) \right)e^{-k_{dark}*t}\#\mathrm{Eq}. S2 \end{aligned}$$

where $\mathrm{Abs}(\mathrm{rec})$is the absorbance after complete recovery, $\mathrm{Abs}(\mathrm{PSS})$is the absorbance at the photostationary state.

At the photostationary state, the population of the *linear* state reaches equilibrium ($\frac{dN_{linear}}{dt}=0$). Under this condition,

$$N_{linear}\left( \infty\right)=\frac{k_{2}}{k_{1}+k_{2}}N_{0}$$

Since $N_{\mathrm{linear}}(0)=xN_{0}$, the normalized absorbance at the photostationary state can be expressed as

$$\begin{aligned} PSS=\frac{Abs.(ꝏ)}{Abs.(0)}=\frac{N_{linear}\left( \infty\right)}{N_{linear}\left( 0 \right)}=\frac{k_{2}}{{x(k}_{1}+k_{2})}=\frac{k_{dark}}{{xk}_{photo}}\#\mathrm{Eq}. S3 \end{aligned}$$

**2. Supplementary Figures and Tables**

**2.1 Characterizations and DFT computational results**

**Figure S1.** Photographs of the dialysis experiment for DASA-2-HMDI-PTMEG_1:1_ in THF at 0, 2, and 9 h (dialysis membrane molecular weight cutoff: 4000 Da).

**Figure S2.** RDG analyses of the interaction between a) *linear* and b) *cyclic* DASA-2 with carbamate moiety (representing PUs hard segment). c) IGMH (top) maps and RDG (bottom) analyses of the interaction between *linear* DASA-2 with Et_2_O (representing PUs soft segment).

**Figure S3.** a) ATR-FTIR spectra of HMDI-PTMEG_1:1_ doped with different concentrations of DASA-2, as well as b) their deconvolution analyses.

**Table S1.** Assignments for the deconvoluted subpeaks of the C=O absorption bands in HMDI-PTMEG_1:1_ doped with different concentrations of DASA-2.

| DASA-2 concentration (wt%) | Wavenumber (cm^-1^)/Area (%) | | |
| --- | --- | --- | --- |
|  | Free ν_C=O_ | Hydrogen bonds | |
|  |  | Disordered | Ordered |
| 0 | 1719/37.9 | 1699/52.3 | 1664/9.75 |
| 0.2 | 1719/36.0 | 1697/54.8 | 1665/9.28 |
| 1 | 1719/36.0 | 1698/57.8 | 1663/6.15 |
| 4 | 1720/27.4 | 1699/66.2 | 1664/6.40 |

**Figure S4.** Deconvolution analyses for the ATR-FTIR spectra of a) Valid-O and b) Valid-A. c) The effect of chain extender on $k_{photo}$ (top) and $k_{dark}$ (bottom) of DASA-2 in HMDI-PTMEG_1:1_.

**Table S2.** Assignments for the deconvoluted subpeaks of the N-H absorption bands in validation PUs.

| Sample | Wavenumber (cm^-1^)/Area (%) | | |
| --- | --- | --- | --- |
|  | Free ν_N-H_ | Hydrogen bonds | |
|  |  | N-H group-hard segment | N-H group-soft segment |
| Valid-O | 3450/3.08 | 3417/2.84 | 3323/94.08 |
| Valid-A | 3449/1.41 | 3407/0.602 | 3326/97.98 |

**Table S3.** Relative ground a state electronic and Gibbs free energies (in kcal mol^-1^) of twisted intermediate species (A' and A''), transition states (TS_1_ and TS_2_) and *cyclic* B in chloroform.

|  | Free DASA-2 | | Covalently incorporated DASA-2 | |
| --- | --- | --- | --- | --- |
|  | △E | △G | △E | △G |
| A' | 0 | 0 | 0 | 0 |
| TS_1_ | 14.3 | 14.0 | 13.3 | 12.5 |
| A'' | 3.3 | 4.0 | 3.3 | 6.1 |
| TS_2_ | 16.6 | 17.4 | 15.3 | 15.8 |
| B | -1.8 | 1.5 | -5.3 | -2.6 |

**Figure S5.** Photographs of the quartz/chromium photomasks used for mask lithography.

**Figure S6.** UV-Vis absorption spectra of a) physically doped DASA-2@HMDI-PTMEG_1:1_ and b) covalently incorporated DASA-2-HMDI-PTMEG_1:1_ before and after UV irradiation.

**Figure S7.** Gel permeation chromatography (GPC) traces of covalently incorporated DASA-2-HMDI-PTMEG_1:1_ before and after UV irradiation.

**Figure S8.** Comparison of the photostationary state of four encoding units.

**Figure S9.** Schematic diagram of preparation and practical application of three-dimensional dual-track optical encryption enable by FF and SS encoding units.

**Table S4.** Representative summary of reversible photochromism kinetics of selective literature-reported DASA compounds in polymer matrices.

| **Ref.** | **Sample** | **Photo-bleaching rate (**$\boldsymbol{k}_{\boldsymbol{p}\boldsymbol{hoto}}$**, s^-1^)** | **Thermal-recovery rate (**$\boldsymbol{k}_{\boldsymbol{dark}}$**, s^-1^)** | **Control parameters of polymer matrices** |
| --- | --- | --- | --- | --- |
| *ACS Macro Lett.* **2017**, *6*, 738−742 (**ref. S1**) | PEMA, *T*_g_ = 65 ℃ | 0.00250 | 0.0000366 (40 ℃) | Glass-transition temperature (*T*_g_) |
|  | PBMA, *T*_g_ = 20 ℃ | 0.00383 | 0.000164 (40 ℃) |  |
| *Polym. Chem.* **2019**, *10*, 6515–6522 (**ref. S2**)[7] | pMAEA_101_ | 0.000263 | 0.000111 (25 ℃) | Polymerization degree |
|  | pMAEA_23_ | 0.000158 | 0.000110 (25 ℃) |  |
|  | pMAEMA_92_ | 0.000321 | 0.000112 (25 ℃) |  |
|  | pMAEMA_29_ | 0.000550 | 0.000112 (25 ℃) |  |
| *Macromol. Rapid Commun.* **2022**, *43*, 2200120 (**ref. S3**) | C4-barb, *n* ~ 40 | 0.0304 | 0.00137 (24 ℃) | Architectural configurations (C: sidechain, M: monoendfunctional, and T: telechelic) |
|  | M2-barb, *n* ~ 110 | 0.0340 | 0.00133 (24 ℃) |  |
|  | T1-barb, *n* ~ 60 | 0.0304 | 0.00137 (24 ℃) |  |
|  | M1-barb, *n* ~15 | 0.0330 | 0.00132 (24 ℃) | Polymerization degree of monoendfunctional PDMS |
|  | C5-barb, *n* ~ 1000 | 0.0340 | 0.00160 (24 ℃) |  |

| **Ref.** | **Sample** | **Photo-bleaching rate (**$\boldsymbol{k}_{\boldsymbol{p}\boldsymbol{hoto}}$**, s^-1^)** | **Thermal-recovery rate (**$\boldsymbol{k}_{\boldsymbol{dark}}$**, s^-1^)** | **Control parameters of polymer matrices** |
| --- | --- | --- | --- | --- |
| *ChemPhotoChem* **2022**, *6*, e202200076 (**ref. S4**) | poly(butyl acrylate) co poly(methyl methacrylate), *T*_g_ = -8.5 ℃ | 0.107 | 0.16 (23 ℃) | Glass-transition temperature |
|  | poly(vinyl acetate) co poly(butyl acrylate) co poly(tertbutyl methacrylate), *T*_g_ = 18 ℃ | 0.104 | 0.046 (23 ℃) |  |
|  | poly(tert-butyl methacrylate) co poly(butyl acrylate), *T*_g_ = -15 ℃ | 0.175 | 0.17 (23 ℃) |  |
|  | plasticized poly(vinyl chloride), *T*_g_ = 1.5 ℃ | 0.107 | 0.12 (23 ℃) |  |
|  | poly(vinyl acetate), *T*_g_ = 35 ℃ | 0.023 | 0.00029 (23 ℃) |  |
| *ACS Appl. Polym. Mater.* **2022**, *4*, 141–149 (**ref. S5**) | 1mol%DASA-PHMA, *T*_g_ = 2 ℃ | 0.135 | 0.00165 (22 ℃) | Glass-transition temperature changed by DASA concentration |
|  | 4mol%DASA-PHMA, *T*_g_ = 4 ℃ | 0.066 | 0.000424 (22 ℃) |  |
|  | 9mol%DASA-PHMA, *T*_g_ = 9 ℃ | 0.0271 | 0.000243 (22 ℃) |  |

| **Ref.** | **Sample** | **Photo-bleaching rate (**$\boldsymbol{k}_{\boldsymbol{p}\boldsymbol{hoto}}$**, s^-1^)** | **Thermal-recovery rate (**$\boldsymbol{k}_{\boldsymbol{dark}}$**, s^-1^)** | **Control parameters of polymer matrices** |
| --- | --- | --- | --- | --- |
| *Chem. Eng. J.* **2022**, *450*, 138090 **(ref. S6**) | DASA:PCL = 1:1 (w/w) | 0.00464 | 0.00884 (60 ℃) | DASA concentration |
|  | DASA:PCL = 1:3 (w/w) | 0.00696 | 0.0108 (60 ℃) |  |
|  | DASA:PCL = 1:5 (w/w) | 0.0150 | 0.00991 (60 ℃) |  |
|  | DASA:PCL = 1:7 (w/w) | 0.0245 | 0.0109 (60 ℃) |  |
|  | PVA+HH | 0.009 | 0.013 (60 ℃) | Molecular ink with ester functional  groups |
|  | PVA+HB | 0.052 | 0.011 (60 ℃) |  |
|  | PVA+PB | 0.018 | 0.010 (60 ℃) |  |
|  | PVA+SA | 0.019 | 0.015 (60 ℃) |  |
|  | PVA+1-OD | 0.005 | 0.002 (60 ℃) |  |
|  | PVA+4-BP | 0.004 | 0.003 (60 ℃) |  |
|  | PVA+BA | 0.012 | 0.003 (60 ℃) |  |
|  | PVA+OTP | 0.012 | 0.007 (60 ℃) |  |
|  | PVA+BP | 0.017 | 0.018 (60 ℃) |  |
|  | PVA+OPD | 0.003 | 0.002 (60 ℃) |  |
| **Ref.** | **Sample** | **Photo-bleaching rate (**$\boldsymbol{k}_{\boldsymbol{p}\boldsymbol{hoto}}$**, s^-1^)** | **Thermal-recovery rate (**$\boldsymbol{k}_{\boldsymbol{dark}}$**, s^-1^)** | **Control parameters of polymer matrices** |
| *Chem. Eng. J.* **2024**, *499*, 156196 **(ref. S7**) | Initial Abs.=1.047 a.u. | 0.1349 | 0.003401 (33.2 °C) | Initial absorption of DASA-PCL films |
|  | Initial Abs.=1.329 a.u. | 0.1110 | 0.003557 (33.2 °C) |  |
|  | Initial Abs.=1.505 a.u. | 0.09499 | 0.003516 (33.2 °C) |  |
|  | Initial Abs.=1.747 a.u. | 0.08458 | 0.003605 (33.2 °C) |  |

**2.2 Time-dependent UV-Vis spectra**

As highlighted in our previous work,[1] meaningful comparison of $k_{photo}$ requires measurements to be performed at comparable initial absorbance levels. However, DASAs intrinsically exist as an equilibrium mixture of *linear* and *cyclic* isomers, and the population ratio between these two states is strongly dependent on the polymer matrix and sample preparation conditions. As a result, identical DASA loadings do not necessarily yield identical film absorbance. Accordingly, the kinetic comparison does not assume identical initial populations of the *linear* state. Instead, the experimentally measured initial absorbance, which represents the population of the colored *linear* species under the given measurement conditions, was used as the normalization parameter for comparing the photo-bleaching kinetics.

Therefore, for the physically dispersed DASA-2@PU systems, $k_{photo}$ values using films prepared with different DASA loadings. For the covalently incorporated DASA-2-PU systems, where the DASA-2 content was fixed by the polymer composition, films with different absorbance values were prepared by varying the casting thickness. The resulting $k_{\mathrm{photo}}$values were subsequently fitted using an empirical absorbance-rate relationship to obtain $k_{\mathrm{photo}}$values at equivalent absorbance levels. In contrast, $k_{dark}$ values were reported as the average of multiple independent measurements.

**Figure S10.** a) Time-resolved UV-Vis absorption spectra of DASA-2@HMDI-PTMEG_1:1_ at $\lambda_{\max}$ 589 nm under white light irradiation and darkness. Exponential fit of absorbance measurements at $\lambda_{\max}$ under b) white light irradiation and c) darkness, where the average $k_{dark}$ is 0.00621 s^-1^. d) The variation of $k_{photo}$ in DASA-2@HMDI-PTMEG_1:1_ with initial absorbance ($y=-0.957x+4.20$, R^2^＞0.98).

**Table S5.** Initial absorbance and reversible photochromic rate constants ($k_{photo}$ and $k_{dark}$) of DASA-2@HMDI-PTMEG_1:1_.

| Number | Initial Abs. (a.u.) | $k_{photo}$ (*10^2^ s^-1^) | R^2^ | $k_{dark}$ (*10^3^ s^-1^) | R^2^ |
| --- | --- | --- | --- | --- | --- |
| 1 | 1.51 | 2.75 | ＞0.99 | 6.32 | ＞0.99 |
| 2 | 1.42 | 2.83 | ＞0.99 | 6.19 | ＞0.99 |
| 3 | 1.33 | 2.95 | ＞0.99 | 6.24 | ＞0.99 |
| 4 | 1.25 | 3.02 | ＞0.99 | 6.15 | ＞0.99 |
| 5 | 1.17 | 3.08 | ＞0.99 | 6.16 | ＞0.99 |

**Figure S11.** a) Time-resolved UV-Vis absorption spectra of DASA-2-HMDI-PTMEG_1:1_ at $\lambda_{\max}$ 588 nm under white light irradiation and darkness. Exponential fit of absorbance measurements at $\lambda_{\max}$ under b) white light irradiation and c) darkness, where the average $k_{dark}$ is 0.00244 s^-1^. d) The variation of $k_{photo}$ in DASA-2-HMDI-PTMEG_1:1_ with initial absorbance ($y=-2.30x+10.1$, R^2^＞0.98).

**Table S6.** Initial absorbance and reversible photochromic rate constants ($k_{photo}$ and $k_{dark}$) of DASA-2-HMDI-PTMEG_1:1_.

| Number | Initial Abs. (a.u.) | $k_{photo}$ (*10^2^ s^-1^) | R^2^ | $k_{dark}$ (*10^3^ s^-1^) | R^2^ |
| --- | --- | --- | --- | --- | --- |
| 1 | 1.55 | 6.50 | ＞0.99 | 2.42 | ＞0.99 |
| 2 | 1.49 | 6.67 | ＞0.99 | 2.42 | ＞0.99 |
| 3 | 1.41 | 6.89 | ＞0.99 | 2.44 | ＞0.99 |
| 4 | 1.35 | 6.99 | ＞0.99 | 2.45 | ＞0.99 |
| 5 | 1.29 | 7.11 | ＞0.99 | 2.47 | ＞0.99 |

**Figure S12.** a) Time-resolved UV-Vis absorption spectra of DASA-2@HMDI-PTMEG_5:1_ at $\lambda_{\max}$ 589 nm under white light irradiation and darkness. Exponential fit of absorbance measurements at $\lambda_{\max}$ under b) white light irradiation and c) darkness, where the average $k_{dark}$ is 0.00728 s^-1^. d) The variation of $k_{photo}$ in DASA-2@HMDI-PTMEG_5:1_ with initial absorbance ($y=-0.0206x+0.0647$, R^2^＞0.99).

**Table S7.** Initial absorbance and reversible photochromic rate constants ($k_{photo}$ and $k_{dark}$) of DASA-2@HMDI-PTMEG_5:1_.

| Number | Initial Abs. (a.u.) | $k_{photo}$ (*10^2^ s^-1^) | R^2^ | $k_{dark}$ (*10^3^ s^-1^) | R^2^ |
| --- | --- | --- | --- | --- | --- |
| 1 | 1.26 | 3.90 | ＞0.99 | 7.31 | ＞0.99 |
| 2 | 0.979 | 4.42 | ＞0.99 | 7.30 | ＞0.99 |
| 3 | 0.872 | 4.64 | ＞0.99 | 7.28 | ＞0.99 |
| 4 | 0.778 | 4.88 | ＞0.99 | 7.27 | ＞0.99 |
| 5 | 0.677 | 5.10 | ＞0.99 | 7.22 | ＞0.99 |

**Figure S13.** a) Time-resolved UV-Vis absorption spectra of DASA-2@HMDI-PTMEG_2:1_ at $\lambda_{\max}$ 589 nm under white light irradiation and darkness. Exponential fit of absorbance measurements at $\lambda_{\max}$ under b) white light irradiation and c) darkness, where the average $k_{dark}$ is 0.00662 s^-1^. d) The variation of $k_{photo}$ in DASA-2@HMDI-PTMEG_2:1_ with initial absorbance ($y=-1.18x+4.53$, R^2^＞0.99).

**Table S8.** Initial absorbance and reversible photochromic rate constants ($k_{photo}$ and $k_{dark}$) of DASA-2@HMDI-PTMEG_2:1_.

| Number | Initial Abs. (a.u.) | $k_{photo}$ (*10^2^ s^-1^) | R^2^ | $k_{dark}$ (*10^3^ s^-1^) | R^2^ |
| --- | --- | --- | --- | --- | --- |
| 1 | 1.67 | 2.58 | ＞0.99 | 6.58 | ＞0.99 |
| 2 | 1.59 | 2.66 | ＞0.99 | 6.62 | ＞0.99 |
| 3 | 1.49 | 2.76 | ＞0.99 | 6.50 | ＞0.99 |
| 4 | 1.40 | 2.89 | ＞0.99 | 6.73 | ＞0.99 |
| 5 | 1.32 | 2.99 | ＞0.99 | 6.69 | ＞0.99 |

**Figure S14.** a) Time-resolved UV-Vis absorption spectra of DASA-2@HMDI-PTMEG_5:7_ at $\lambda_{\max}$ 589 nm under white light irradiation and darkness. Exponential fit of absorbance measurements at $\lambda_{\max}$ under b) white light irradiation and c) darkness, where the average $k_{dark}$ is 0.00564 s^-1^. d) The variation of $k_{photo}$ in DASA-2@HMDI-PTMEG_5:7_ with initial absorbance ($y=-1.47x+4.07$, R^2^＞0.96).

**Table S9.** Initial absorbance and reversible photochromic rate constants ($k_{photo}$ and $k_{dark}$) of DASA-2@HMDI-PTMEG_5:7_.

| Number | Initial Abs. (a.u.) | $k_{photo}$ (*10^2^ s^-1^) | R^2^ | $k_{dark}$ (*10^3^ s^-1^) | R^2^ |
| --- | --- | --- | --- | --- | --- |
| 1 | 1.20 | 2.343 | ＞0.99 | 5.70 | ＞0.99 |
| 2 | 1.05 | 2.48 | ＞0.99 | 5.65 | ＞0.99 |
| 3 | 0.928 | 2.70 | ＞0.99 | 5.63 | ＞0.99 |
| 4 | 0.827 | 2.81 | ＞0.99 | 5.62 | ＞0.99 |
| 5 | 0.729 | 3.05 | ＞0.99 | 5.60 | ＞0.99 |

**Figure S15.** a) Time-resolved UV-Vis absorption spectra of DASA-2@HMDI-PTMEG_1:2_ at $\lambda_{\max}$ 587 nm under white light irradiation and darkness. Exponential fit of absorbance measurements at $\lambda_{\max}$ under b) white light irradiation and c) darkness, where the average $k_{dark}$ is 0.00458 s^-1^. d) The variation of $k_{photo}$ in DASA-2@HMDI-PTMEG_1:2_ with initial absorbance ($y=-1.36x+3.59$, R^2^＞0.98).

**Table S10.** Initial absorbance and reversible photochromic rate constants ($k_{photo}$ and $k_{dark}$) of DASA-2@HMDI-PTMEG_1:2_.

| Number | Initial Abs. (a.u.) | $k_{photo}$ (*10^2^ s^-1^) | R^2^ | $k_{dark}$ (*10^3^ s^-1^) | R^2^ |
| --- | --- | --- | --- | --- | --- |
| 1 | 1.54 | 1.50 | ＞0.99 | 4.65 | ＞0.99 |
| 2 | 1.36 | 1.72 | ＞0.99 | 4.63 | ＞0.99 |
| 3 | 1.20 | 2.00 | ＞0.99 | 4.56 | ＞0.99 |
| 4 | 1.06 | 2.19 | ＞0.99 | 4.53 | ＞0.99 |
| 5 | 0.921 | 2.31 | ＞0.99 | 4.55 | ＞0.99 |

**Figure S16.** a) Time-resolved UV-Vis absorption spectra of DASA-2@HMDI-PTMEG_1:3_ at $\lambda_{\max}$ 588 nm under white light irradiation and darkness. Exponential fit of absorbance measurements at $\lambda_{\max}$ under b) white light irradiation and c) darkness, where the average $k_{dark}$ is 0.00359 s^-1^. d) The variation of $k_{photo}$ in DASA-2@HMDI-PTMEG_1:3_ with initial absorbance ($y=-0.603x+2.16$, R^2^＞0.99).

**Table S11.** Initial absorbance and reversible photochromic rate constants ($k_{photo}$ and $k_{dark}$) of DASA-2@HMDI-PTMEG_1:3_.

| Number | Initial Abs. (a.u.) | $k_{photo}$ (*10^2^ s^-1^) | R^2^ | $k_{dark}$ (*10^3^ s^-1^) | R^2^ |
| --- | --- | --- | --- | --- | --- |
| 1 | 1.72 | 1.13 | ＞0.99 | 3.67 | ＞0.99 |
| 2 | 1.57 | 1.20 | ＞0.99 | 3.58 | ＞0.99 |
| 3 | 1.41 | 1.30 | ＞0.99 | 3.54 | ＞0.99 |
| 4 | 1.29 | 1.38 | ＞0.99 | 3.60 | ＞0.99 |
| 5 | 1.17 | 1.46 | ＞0.99 | 3.57 | ＞0.99 |

**Figure S17.** a) Time-resolved UV-Vis absorption spectra of DASA-2@Valid-O at $\lambda_{\max}$ 589 nm under white light irradiation and darkness. Exponential fit of absorbance measurements at $\lambda_{\max}$ under b) white light irradiation and c) darkness, where the average $k_{dark}$ is 0.00641 s^-1^. d) The variation of $k_{photo}$ in DASA-2@Valid-O with initial absorbance ($y=-0.935x+4.24$, R^2^＞0.99).

**Table S12.** Initial absorbance and reversible photochromic rate constants ($k_{photo}$ and $k_{dark}$) of DASA-2@Valid-O.

| Number | Initial Abs. (a.u.) | $k_{photo}$ (*10^2^ s^-1^) | R^2^ | $k_{dark}$ (*10^3^ s^-1^) | R^2^ |
| --- | --- | --- | --- | --- | --- |
| 1 | 1.64 | 2.71 | ＞0.99 | 6.54 | ＞0.99 |
| 2 | 1.56 | 2.78 | ＞0.99 | 6.52 | ＞0.99 |
| 3 | 1.47 | 2.87 | ＞0.99 | 6.35 | ＞0.99 |
| 4 | 1.41 | 2.93 | ＞0.99 | 6.32 | ＞0.99 |
| 5 | 1.35 | 2.98 | ＞0.99 | 6.34 | ＞0.99 |

**Figure S18.** a) Time-resolved UV-Vis absorption spectra of DASA-2@Valid-A at $\lambda_{\max}$ 587 nm under white light irradiation and darkness. Exponential fit of absorbance measurements at $\lambda_{\max}$ under b) white light irradiation and c) darkness, where the average $k_{dark}$ is 0.00573 s^-1^. d) The variation of $k_{photo}$ in DASA-2@Valid-A with initial absorbance ($y=-0.473x+1.59$, R^2^＞0.98).

**Table S13.** Initial absorbance and reversible photochromic rate constants ($k_{photo}$ and $k_{dark}$) of DASA-2@Valid-A.

| Number | Initial Abs. (a.u.) | $k_{photo}$ (*10^2^ s^-1^) | R^2^ | $k_{dark}$ (*10^3^ s^-1^) | R^2^ |
| --- | --- | --- | --- | --- | --- |
| 1 | 1.88 | 0.718 | ＞0.99 | 5.94 | ＞0.99 |
| 2 | 1.41 | 0.914 | ＞0.99 | 5.87 | ＞0.99 |
| 3 | 1.01 | 1.07 | ＞0.99 | 5.78 | ＞0.99 |
| 4 | 0.690 | 1.27 | ＞0.99 | 5.62 | ＞0.99 |
| 5 | 0.457 | 1.39 | ＞0.99 | 5.46 | ＞0.99 |

**Figure S19.** Exponential fit of absorbance measurements of DASA-2 monomer at $\lambda_{\max}$ 587 nm and DASA-2-HMDI-PTMEG_1:1_ at $\lambda_{\max}$ 588 nm in chloroform solution under a) white light irradiation and b) darkness.

**Table S14.** Initial absorbance and reversible photochromic rate constants ($k_{photo}$ and $k_{dark}$) of DASA-2 monomer and DASA-2-HMDI-PTMEG_1:1_ in chloroform solution.

| Sample | Initial Abs. (a.u.) | $k_{photo}$  (*10^2^ s^-1^) | R^2^ | $k_{dark}$  (*10^3^ s^-1^) | R^2^ |
| --- | --- | --- | --- | --- | --- |
| DASA-2 monomer | 0.993 | 20.4 | ＞0.99 | 14.6 | ＞0.99 |
| DASA-2-HMDI-PTMEG_1:1_ | 0.992 | 49.3 | ＞0.99 | 4.55 | ＞0.99 |

**Figure S20.** a) Time-resolved UV-Vis absorption spectra of DASA-2@HMDI-PCL_2:1_ at $\lambda_{\max}$ 591 nm under white light irradiation and darkness. Exponential fit of absorbance measurements at $\lambda_{\max}$ under b) white light irradiation and c) darkness, where the average $k_{dark}$ is 0.00466 s^-1^. d) The variation of $k_{photo}$ in DASA-2@HMDI-PCL_2:1_ with initial absorbance ($y=-0.589x+2.83$, R^2^＞0.99).

**Table S15.** Initial absorbance and reversible photochromic rate constants ($k_{photo}$ and $k_{dark}$) of DASA-2@ HMDI-PCL_2:1_.

| Number | Initial Abs. (a.u.) | $k_{photo}$ (*10^2^ s^-1^) | R^2^ | $k_{dark}$ (*10^3^ s^-1^) | R^2^ |
| --- | --- | --- | --- | --- | --- |
| 1 | 1.44 | 1.97 | ＞0.99 | 4.79 | ＞0.99 |
| 2 | 1.33 | 2.05 | ＞0.99 | 4.69 | ＞0.99 |
| 3 | 1.25 | 2.08 | ＞0.99 | 4.64 | ＞0.99 |
| 4 | 1.16 | 2.15 | ＞0.99 | 4.59 | ＞0.99 |
| 5 | 1.10 | 2.18 | ＞0.99 | 4.58 | ＞0.99 |

**Figure S21.** a) Time-resolved UV-Vis absorption spectra of DASA-2@HMDI-PCL_1:1_ at $\lambda_{\max}$ 589 nm under white light irradiation and darkness. Exponential fit of absorbance measurements at $\lambda_{\max}$ under b) white light irradiation and c) darkness, where the average $k_{dark}$ is 0.00425 s^-1^. d) The variation of $k_{photo}$ in DASA-2@HMDI-PCL_1:1_ with initial absorbance ($y=-0.705x+2.38$, R^2^＞0.97).

**Table S16.** Initial absorbance and reversible photochromic rate constants ($k_{photo}$ and $k_{dark}$) of DASA-2@ HMDI-PCL_1:1_.

| Number | Initial Abs. (a.u.) | $k_{photo}$ (*10^2^ s^-1^) | R^2^ | $k_{dark}$ (*10^3^ s^-1^) | R^2^ |
| --- | --- | --- | --- | --- | --- |
| 1 | 1.50 | 1.35 | ＞0.99 | 4.33 | ＞0.99 |
| 2 | 1.31 | 1.43 | ＞0.99 | 4.24 | ＞0.99 |
| 3 | 1.11 | 1.58 | ＞0.99 | 4.29 | ＞0.99 |
| 4 | 0.937 | 1.75 | ＞0.99 | 4.21 | ＞0.99 |
| 5 | 0.791 | 1.82 | ＞0.99 | 4.17 | ＞0.99 |

**Figure S22.** a) Time-resolved UV-Vis absorption spectra of DASA-2@HMDI-PCL_1:2_ at $\lambda_{\max}$ 589 nm under white light irradiation and darkness. Exponential fit of absorbance measurements at $\lambda_{\max}$ under b) white light irradiation and c) darkness, where the average $k_{dark}$ is 0.00405 s^-1^. d) The variation of $k_{photo}$ in DASA-2@HMDI-PCL_1:2_ with initial absorbance ($y=-0.355x+1.98$, R^2^＞0.92).

**Table S17.** Initial absorbance and reversible photochromic rate constants ($k_{photo}$ and $k_{dark}$) of DASA-2@ HMDI-PCL_1:2_.

| Number | Initial Abs. (a.u.) | $k_{photo}$ (*10^2^ s^-1^) | R^2^ | $k_{dark}$ (*10^3^ s^-1^) | R^2^ |
| --- | --- | --- | --- | --- | --- |
| 1 | 1.33 | 1.51 | ＞0.99 | 4.07 | ＞0.99 |
| 2 | 1.16 | 1.58 | ＞0.99 | 4.07 | ＞0.99 |
| 3 | 1.06 | 1.60 | ＞0.99 | 4.07 | ＞0.99 |
| 4 | 0.902 | 1.64 | ＞0.99 | 4.04 | ＞0.99 |
| 5 | 0.835 | 1.71 | ＞0.99 | 4.00 | ＞0.99 |

**Figure S23.** a) Time-resolved UV-Vis absorption spectra of DASA-2@HMDI-PCL_1:3_ at $\lambda_{\max}$ 588 nm under white light irradiation and darkness. Exponential fit of absorbance measurements at $\lambda_{\max}$ under b) white light irradiation and c) darkness, where the average $k_{dark}$ is 0.00388 s^-1^. d) The variation of $k_{photo}$ in DASA-2@HMDI-PCL_1:3_ with initial absorbance ($y=-0.379x+1.59$, R^2^＞0.86).

**Table S18.** Initial absorbance and reversible photochromic rate constants ($k_{photo}$ and $k_{dark}$) of DASA-2@ HMDI-PCL_1:3_.

| Number | Initial Abs. (a.u.) | $k_{photo}$ (*10^2^ s^-1^) | R^2^ | $k_{dark}$ (*10^3^ s^-1^) | R^2^ |
| --- | --- | --- | --- | --- | --- |
| 1 | 2.01 | 0.836 | ＞0.99 | 3.96 | ＞0.99 |
| 2 | 1.85 | 0.879 | ＞0.99 | 3.97 | ＞0.99 |
| 3 | 1.70 | 0.914 | ＞0.99 | 3.84 | ＞0.99 |
| 4 | 1.56 | 1.02 | ＞0.99 | 3.75 | ＞0.99 |

**Figure S24.** a) Time-resolved UV-Vis absorption spectra of DASA-2@IPDI-PTMEG_1:1_ at $\lambda_{\max}$ 587 nm under white light irradiation and darkness. Exponential fit of absorbance measurements at $\lambda_{\max}$ under b) white light irradiation and c) darkness, where the average $k_{dark}$ is 0.00660 s^-1^. d) The variation of $k_{photo}$ in DASA-2@IPDI-PTMEG_1:1_ with initial absorbance ($y=-0.921x+2.34$, R^2^＞0.99).

**Table S19.** Initial absorbance and reversible photochromic rate constants ($k_{photo}$ and $k_{dark}$) of DASA-2@IPDI-PTMEG_1:1_.

| Number | Initial Abs. (a.u.) | $k_{photo}$ (*10^2^ s^-1^) | R^2^ | $k_{dark}$ (*10^3^ s^-1^) | R^2^ |
| --- | --- | --- | --- | --- | --- |
| 1 | 1.32 | 1.16 | ＞0.99 | 6.80 | ＞0.99 |
| 2 | 1.22 | 1.22 | ＞0.99 | 6.78 | ＞0.99 |
| 3 | 1.12 | 1.29 | ＞0.99 | 6.69 | ＞0.99 |
| 4 | 1.03 | 1.35 | ＞0.99 | 6.67 | ＞0.99 |
| 5 | 0.299 | 2.07 | ＞0.99 | 6.06 | ＞0.99 |

**Figure S25.** a) Time-resolved UV-Vis absorption spectra of DASA-2@IPDI-PTMEG_1:2_ at $\lambda_{\max}$ 587 nm under white light irradiation and darkness. Exponential fit of absorbance measurements at $\lambda_{\max}$ under b) white light irradiation and c) darkness, where the average $k_{dark}$ is 0.00546 s^-1^. d) The variation of $k_{photo}$ in DASA-2@IPDI-PTMEG_1:2_ with initial absorbance ($y=-0.641x+1.87$, R^2^＞0.96).

**Table S20.** Initial absorbance and reversible photochromic rate constants ($k_{photo}$ and $k_{dark}$) of DASA-2@IPDI-PTMEG_1:2_.

| Number | Initial Abs. (a.u.) | $k_{photo}$ (*10^2^ s^-1^) | R^2^ | $k_{dark}$ (*10^3^ s^-1^) | R^2^ |
| --- | --- | --- | --- | --- | --- |
| 1 | 0.966 | 1.24 | ＞0.99 | 5.39 | ＞0.99 |
| 2 | 0.914 | 1.31 | ＞0.99 | 5.50 | ＞0.99 |
| 3 | 0.829 | 1.33 | ＞0.99 | 5.36 | ＞0.99 |
| 4 | 0.754 | 1.38 | ＞0.99 | 5.52 | ＞0.99 |
| 5 | 0.658 | 1.45 | ＞0.99 | 5.55 | ＞0.99 |

**Figure S26.** a) Time-resolved UV-Vis absorption spectra of DASA-2@IPDI-PTMEG_1:3_ at $\lambda_{\max}$ 587 nm under white light irradiation and darkness. Exponential fit of absorbance measurements at $\lambda_{\max}$ under b) white light irradiation and c) darkness, where the average $k_{dark}$ is 0.00384 s^-1^. d) The variation of $k_{photo}$ in DASA-2@IPDI-PTMEG_1:3_ with initial absorbance ($y=-0.463x+1.48$, R^2^＞0.98).

**Table S21.** Initial absorbance and reversible photochromic rate constants ($k_{photo}$ and $k_{dark}$) of DASA-2@IPDI-PTMEG_1:3_.

| Number | Initial Abs. (a.u.) | $k_{photo}$ (*10^2^ s^-1^) | R^2^ | $k_{dark}$ (*10^3^ s^-1^) | R^2^ |
| --- | --- | --- | --- | --- | --- |
| 1 | 1.61 | 0.741 | ＞0.99 | 3.97 | ＞0.99 |
| 2 | 1.46 | 0.796 | ＞0.99 | 3.90 | ＞0.99 |
| 3 | 1.34 | 0.846 | ＞0.99 | 3.79 | ＞0.99 |
| 4 | 1.23 | 0.916 | ＞0.99 | 3.76 | ＞0.99 |
| 5 | 1.12 | 0.964 | ＞0.99 | 3.77 | ＞0.99 |

**Figure S27.** a) Time-resolved UV-Vis absorption spectra of DASA-2@IPDI-PTMEG_1:5_ at $\lambda_{\max}$ 587 nm under white light irradiation and darkness. Exponential fit of absorbance measurements at $\lambda_{\max}$ under b) white light irradiation and c) darkness, where the average $k_{dark}$ is 0.00206 s^-1^. d) The variation of $k_{photo}$ in DASA-2@IPDI-PTMEG_1:5_ with initial absorbance ($y=-0.317x+1.11$, R^2^＞0.98).

**Table S22.** Initial absorbance and reversible photochromic rate constants ($k_{photo}$ and $k_{dark}$) of DASA-2@IPDI-PTMEG_1:5_.

| Number | Initial Abs. (a.u.) | $k_{photo}$ (*10^2^ s^-1^) | R^2^ | $k_{dark}$ (*10^3^ s^-1^) | R^2^ |
| --- | --- | --- | --- | --- | --- |
| 1 | 1.06 | 0.769 | ＞0.99 | 2.09 | ＞0.99 |
| 2 | 0.954 | 0.811 | ＞0.99 | 2.08 | ＞0.99 |
| 3 | 0.862 | 0.834 | ＞0.99 | 2.06 | ＞0.99 |
| 4 | 0.772 | 0.862 | ＞0.99 | 2.07 | ＞0.99 |
| 5 | 0.687 | 0.889 | ＞0.99 | 2.01 | ＞0.99 |

**Figure S28.** a) Time-resolved UV-Vis absorption spectra of DASA-2@IPDI-PCL_2:1_ at $\lambda_{\max}$ 589 nm under white light irradiation and darkness. Exponential fit of absorbance measurements at $\lambda_{\max}$ under b) white light irradiation and c) darkness, where the average $k_{dark}$ is 0.00496 s^-1^. d) The variation of $k_{photo}$ in DASA-2@IPDI-PCL_2:1_ with initial absorbance ($y=-0.836x+2.13$, R^2^＞0.99).

**Table S23.** Initial absorbance and reversible photochromic rate constants ($k_{photo}$ and $k_{dark}$) of DASA-2@IPDI-PCL_2:1_.

| Number | Initial Abs. (a.u.) | $k_{photo}$ (*10^2^ s^-1^) | R^2^ | $k_{dark}$ (*10^3^ s^-1^) | R^2^ |
| --- | --- | --- | --- | --- | --- |
| 1 | 1.50 | 0.868 | ＞0.99 | 5.29 | ＞0.99 |
| 2 | 1.46 | 0.918 | ＞0.99 | 5.19 | ＞0.99 |
| 3 | 0.898 | 1.42 | ＞0.99 | 4.81 | ＞0.99 |
| 4 | 0.834 | 1.43 | ＞0.99 | 4.77 | ＞0.99 |
| 5 | 0.776 | 1.47 | ＞0.99 | 4.72 | ＞0.99 |

**Figure S29.** a) Time-resolved UV-Vis absorption spectra of DASA-2@IPDI-PCL_1:1_ at $\lambda_{\max}$ 589 nm under white light irradiation and darkness. Exponential fit of absorbance measurements at $\lambda_{\max}$ under b) white light irradiation and c) darkness, where the average $k_{dark}$ is 0.00452 s^-1^. d) The variation of $k_{photo}$ in DASA-2@IPDI-PCL_1:1_ with initial absorbance ($y=-0.486x+1.57$, R^2^＞0.99).

**Table S24.** Initial absorbance and reversible photochromic rate constants ($k_{photo}$ and $k_{dark}$) of DASA-2@IPDI-PCL_1:1_.

| Number | Initial Abs. (a.u.) | $k_{photo}$ (*10^2^ s^-1^) | R^2^ | $k_{dark}$ (*10^3^ s^-1^) | R^2^ |
| --- | --- | --- | --- | --- | --- |
| 1 | 1.63 | 0.784 | ＞0.99 | 4.55 | ＞0.99 |
| 2 | 1.34 | 0.914 | ＞0.99 | 4.52 | ＞0.99 |
| 3 | 1.18 | 0.991 | ＞0.99 | 4.50 | ＞0.99 |
| 4 | 1.05 | 1.06 | ＞0.99 | 4.53 | ＞0.99 |
| 5 | 0.907 | 1.14 | ＞0.99 | 4.5 | ＞0.99 |

**Figure S30.** a) Time-resolved UV-Vis absorption spectra of DASA-2@IPDI-PCL_1:2_ at $\lambda_{\max}$ 589 nm under white light irradiation and darkness. Exponential fit of absorbance measurements at $\lambda_{\max}$ under b) white light irradiation and c) darkness, where the average $k_{dark}$ is 0.00408 s^-1^. d) The variation of $k_{photo}$ in DASA-2@IPDI-PCL_1:2_ with initial absorbance ($y=-0.381x+1.37$, R^2^＞0.97).

**Table S25.** Initial absorbance and reversible photochromic rate constants ($k_{photo}$ and $k_{dark}$) of DASA-2@IPDI-PCL_1:2_.

| Number | Initial Abs. (a.u.) | $k_{photo}$ (*10^2^ s^-1^) | R^2^ | $k_{dark}$ (*10^3^ s^-1^) | R^2^ |
| --- | --- | --- | --- | --- | --- |
| 1 | 1.49 | 0.796 | ＞0.99 | 4.10 | ＞0.99 |
| 2 | 1.37 | 0.861 | ＞0.99 | 4.07 | ＞0.99 |
| 3 | 1.31 | 0.877 | ＞0.99 | 4.11 | ＞0.99 |
| 4 | 1.18 | 0.916 | ＞0.99 | 4.05 | ＞0.99 |
| 5 | 1.16 | 0.929 | ＞0.99 | 4.07 | ＞0.99 |

**Figure S31.** a) Time-resolved UV-Vis absorption spectra of DASA-2@IPDI-PCL_1:5_ at $\lambda_{\max}$ 588 nm under white light irradiation and darkness. Exponential fit of absorbance measurements at $\lambda_{\max}$ under b) white light irradiation and c) darkness, where the average $k_{dark}$ is 0.00243 s^-1^. d) The variation of $k_{photo}$ in DASA-2@IPDI-PCL_1:5_ with initial absorbance ($y=-0.265x+1.02$, R^2^＞0.97).

**Table S26.** Initial absorbance and reversible photochromic rate constants ($k_{photo}$ and $k_{dark}$) of DASA-2@IPDI-PCL_1:5_.

| Number | Initial Abs. (a.u.) | $k_{photo}$ (*10^2^ s^-1^) | R^2^ | $k_{dark}$ (*10^3^ s^-1^) | R^2^ |
| --- | --- | --- | --- | --- | --- |
| 1 | 1.28 | 0.679 | ＞0.99 | 2.44 | ＞0.99 |
| 2 | 1.21 | 0.703 | ＞0.99 | 2.49 | ＞0.99 |
| 3 | 1.13 | 0.723 | ＞0.99 | 2.45 | ＞0.99 |
| 4 | 1.17 | 0.707 | ＞0.99 | 2.36 | ＞0.99 |
| 5 | 1.08 | 0.735 | ＞0.99 | 2.40 | ＞0.99 |

**Figure S32.** a) Time-resolved UV-Vis absorption spectra of DASA-2-HMDI-PCL_1:1_ at $\lambda_{\max}$ 589 nm under white light irradiation and darkness. Exponential fit of absorbance measurements at $\lambda_{\max}$ under b) white light irradiation and c) darkness, where the average $k_{dark}$ is 0.00239 s^-1^. d) The variation of $k_{photo}$ in DASA-2-HMDI-PCL_1:1_ with initial absorbance ($y=-1.92x+7.14$, R^2^＞0.92).

**Table S27.** Initial absorbance and reversible photochromic rate constants ($k_{photo}$ and $k_{dark}$) of DASA-2-HMDI-PCL_1:1_.

| Number | Initial Abs. (a.u.) | $k_{photo}$ (*10^2^ s^-1^) | R^2^ | $k_{dark}$ (*10^3^ s^-1^) | R^2^ |
| --- | --- | --- | --- | --- | --- |
| 1 | 1.24 | 4.80 | ＞0.99 | 2.43 | ＞0.99 |
| 2 | 1.18 | 4.84 | ＞0.99 | 2.41 | ＞0.99 |
| 3 | 1.15 | 4.94 | ＞0.99 | 2.38 | ＞0.99 |
| 4 | 1.13 | 5.03 | ＞0.99 | 2.38 | ＞0.99 |
| 5 | 1.04 | 5.16 | ＞0.99 | 2.36 | ＞0.99 |

**Figure S33.** a) Time-resolved UV-Vis absorption spectra of DASA-2-IPDI-PTMEG_1:1_ at $\lambda_{\max}$ 589 nm under white light irradiation and darkness. Exponential fit of absorbance measurements at $\lambda_{\max}$ under b) white light irradiation and c) darkness, where the average $k_{dark}$ is 0.00347 s^-1^. d) The variation of $k_{photo}$ in DASA-2-IPDI-PTMEG_1:1_ with initial absorbance ($y=-1.58x+5.05$, R^2^＞0.97).

**Table S28.** Initial absorbance and reversible photochromic rate constants ($k_{photo}$ and $k_{dark}$) of DASA-2-IPDI-PTMEG_1:1_.

| Number | Initial Abs. (a.u.) | $k_{photo}$ (*10^2^ s^-1^) | R^2^ | $k_{dark}$ (*10^3^ s^-1^) | R^2^ |
| --- | --- | --- | --- | --- | --- |
| 1 | 0.820 | 3.78 | ＞0.99 | 3.60 | ＞0.99 |
| 2 | 0.778 | 3.80 | ＞0.99 | 3.43 | ＞0.99 |
| 3 | 0.693 | 3.93 | ＞0.99 | 3.44 | ＞0.99 |
| 4 | 0.641 | 4.04 | ＞0.99 | 3.44 | ＞0.99 |
| 5 | 0.590 | 4.13 | ＞0.99 | 3.43 | ＞0.99 |

**Figure S34.** a) Time-resolved UV-Vis absorption spectra of DASA-2-IPDI-PCL_1:1_ at $\lambda_{\max}$ 588 nm under white light irradiation and darkness. Exponential fit of absorbance measurements at $\lambda_{\max}$ under b) white light irradiation and c) darkness, where the average $k_{dark}$ is 0.00295 s^-1^. d) The variation of $k_{photo}$ in DASA-2-IPDI-PCL_1:1_ with initial absorbance ($y=-1.10x+3.85$, R^2^＞0.95).

**Table S29.** Initial absorbance and reversible photochromic rate constants ($k_{photo}$ and $k_{dark}$) of DASA-2-IPDI-PCL_1:1_.

| Number | Initial Abs. (a.u.) | $k_{photo}$ (*10^2^ s^-1^) | R^2^ | $k_{dark}$ (*10^3^ s^-1^) | R^2^ |
| --- | --- | --- | --- | --- | --- |
| 1 | 1.27 | 2.44 | ＞0.99 | 3.04 | ＞0.99 |
| 2 | 1.14 | 2.62 | ＞0.99 | 2.92 | ＞0.99 |
| 3 | 1.11 | 2.63 | ＞0.99 | 2.94 | ＞0.99 |
| 4 | 1.08 | 2.65 | ＞0.99 | 2.91 | ＞0.99 |
| 5 | 1.06 | 2.65 | ＞0.99 | 2.92 | ＞0.99 |

**Figure S35.** a) Time-resolved UV-Vis absorption spectra of DASA-2-HMDI-PCL_1:3_ at $\lambda_{\max}$ 589 nm under white light irradiation and darkness. Exponential fit of absorbance measurements at $\lambda_{\max}$ under b) white light irradiation and c) darkness, where the average $k_{dark}$ is 0.00126 s^-1^. d) The variation of $k_{photo}$ in DASA-2-HMDI-PCL_1:3_ with initial absorbance ($y=-2.65x+6.39$, R^2^＞0.99).

**Table S30.** Initial absorbance and reversible photochromic rate constants ($k_{photo}$ and $k_{dark}$) of DASA-2-HMDI-PCL_1:3_.

| Number | Initial Abs. (a.u.) | $k_{photo}$ (*10^2^ s^-1^) | R^2^ | $k_{dark}$ (*10^3^ s^-1^) | R^2^ |
| --- | --- | --- | --- | --- | --- |
| 1 | 0.895 | 4.02 | ＞0.99 | 1.28 | ＞0.99 |
| 2 | 0.828 | 4.19 | ＞0.99 | 1.28 | ＞0.99 |
| 3 | 0.773 | 4.35 | ＞0.99 | 1.26 | ＞0.99 |
| 4 | 0.725 | 4.45 | ＞0.99 | 1.25 | ＞0.99 |
| 5 | 0.682 | 4.59 | ＞0.99 | 1.25 | ＞0.99 |

**Figure S36.** ATR-FTIR spectra of a) HMDI-PTMEG*_x_*_:_*_y_*, b) validation PUs, c) HMDI-PCL*_x_*_:_*_y_* d) PDI-PTMEG*_x_*_:_*_y_*, e) IPDI-PCL*_x_*_:_*_y_* and f) DASA-covalently incorporated PUs. ν_N-H_ (3319, 3324, 3334, 3326 and 3350 cm^-1^), ν_C=O_ (1724 and 1723 cm^-1^) of PCL, ν_amide II_ (1527, 1525, 1531 and 1528 cm^-1^), and ν_C-O-C_ (1102, 1100 and 1104 cm^-1^) of PTMEG.

**Figure S37.** DSC temperature rise curves of a) HMDI-PTMEG*_x_*_:_*_y_*, b) validation PUs, c) HMDI-PCL*_x_*_:_*_y_* d) PDI-PTMEG*_x_*_:_*_y_*, e) IPDI-PCL*_x_*_:_*_y_* and f) DASA-covalently incorporated PUs. Low crystallinity and a sub-ambient glass transition temperature ensure high matrix transparency for efficient photon utilization and sufficient free volume[4-6] for molecular isomerization in the reversible photochromic processes.

**Table S31.** Molecular weight and polydispersity index of synthetic PUs.

| Sample | $\bar{M}$_n_ (kDa) | $\bar{M}$_w_ (kDa) | Polydispersity index |
| --- | --- | --- | --- |
| HMDI-PTMEG_1:3_ | 40.5 | 60.3 | 1.49 |
| HMDI-PTMEG_1:2_ | 103.8 | 188.6 | 1.82 |
| HMDI-PTMEG_5:7_ | 68.3 | 110.3 | 1.61 |
| HMDI-PTMEG_1:1_ | 97.1 | 175.3 | 1.80 |
| HMDI-PTMEG_2:1_ | 85.6 | 126.5 | 1.48 |
| HMDI-PTMEG_5:1_ | 92.3 | 127.6 | 1.38 |
| Valid-A^a^ | 19.1 | 29.3 | 1.53 |
| Valid-O | 77.3 | 129.9 | 1.68 |
| HMDI-PCL_1:3_ | 99.1 | 187.2 | 1.89 |
| HMDI-PCL_1:2_ | 133.7 | 249.3 | 1.86 |
| HMDI-PCL_1:1_ | 89.5 | 173.2 | 1.93 |
| HMDI-PCL_2:1_ | 74.4 | 131.7 | 1.77 |
| IPDI-PTMEG_1:5_ | 52.2 | 92.3 | 1.77 |
| IPDI-PTMEG_1:3_ | 34.8 | 66.2 | 1.90 |
| IPDI-PTMEG_1:2_ | 64.0 | 105.1 | 1.64 |
| IPDI-PTMEG_1:1_ | 89.9 | 173.9 | 1.94 |
| IPDI-PCL_1:5_ | 71.9 | 124.5 | 1.73 |
| IPDI-PCL_1:2_ | 99.7 | 189.6 | 1.90 |
| IPDI-PCL_1:1_ | 92.7 | 181.8 | 1.96 |
| IPDI-PCL_2:1_ | 86.4 | 166.0 | 1.92 |
| DASA-2-HMDI-PTMEG_1:1_ | 30.3 | 53.4 | 1.76 |
| DASA-2-HMDI-PCL_1:1_ | 26.1 | 45.0 | 1.73 |
| DASA-2-IPDI-PTMEG_1:1_ | 23.6 | 40.6 | 1.72 |
| DASA-2-IPDI-PCL_1:1_ | 16.7 | 26.5 | 1.59 |
| DASA-2-HMDI-PCL_1:3_ | 13.1 | 19.8 | 1.51 |

^a^ Using 0.05 mol L^-1^ DMF/LiBr solution as eluent.

**Table S32.** Thermal properties of synthetic PUs.

| Sample | *T*_g_  (℃) | *T*_m,s_  (℃) | △*H*_m,s_  (J/g) | *T*_m,h_  (℃) | △*H*_m,h_  (J/g) |
| --- | --- | --- | --- | --- | --- |
| HMDI-PTMEG_1:3_ | -78 | / | / |  | / |
| HMDI-PTMEG_1:2_ | -70 | / | / | 96.9 | / |
| HMDI-PTMEG_5:7_ | -74 | 18.7 | 32.7 | / | / |
| HMDI-PTMEG_1:1_ | -69 | / | / | / | / |
| HMDI-PTMEG_2:1_ | -67 | 24.3 | / | / | / |
| HMDI-PTMEG_5:1_ | -60 | 37.9 | 63.7 | / | / |
| Valid-A | -65.5 | / | / | / | / |
| Valid-O | -70.4 | 38.5 | 44.0 | / | / |
| HMDI-PCL_1:3_ | -46.2 | / | / | / | / |
| HMDI-PCL_1:2_ | -44.9 | / | / | 97.2 | 10.04 |
| HMDI-PCL_1:1_ | -47.6 | 39.7 | 11.6 | / | / |
| HMDI-PCL_2:1_ | -43.7 | 43.5 | 37.5 | / | / |
| IPDI-PTMEG_1:5_ | -77 | 36.4 | / | / | / |
| IPDI-PTMEG_1:3_ | -75 | 36.2 | 6.8 | / | / |
| IPDI-PTMEG_1:2_ | -63 | 42.8 | 51.8 | / | / |
| IPDI-PTMEG_1:1_ | -66 | 20.1 | / | / | / |
| IPDI-PCL_1:5_ | -37.5 | 40.3 | / | / | / |
| IPDI-PCL_1:2_ | -36.5 | 39.9 | 23.0 | / | / |
| IPDI-PCL_1:1_ | -41.9 | 38.5 | 26.7 | / | / |
| IPDI-PCL_2:1_ | -43.3 | 39.7 | 40.3 | / | / |
| DASA-2-HMDI-PTMEG_1:1_ | -69.7 | / | / | / | / |
| DASA-2-HMDI-PCL_1:1_ | -39.7 | 42.6 | 34.2 | / | / |
| DASA-2-IPDI-PTMEG_1:1_ | -61.9 | 40.6 | 60.6 | / | / |
| DASA-2-IPDI-PCL_1:1_ | -43.7 | / | / | / | / |
| DASA-2-HMDI-PCL_1:3_ | -46.5 | / | / | / | / |

*T*_g_: glass-transition temperature; *T*_m,s_ and *T*_m,h_: melting temperature of the soft- and hard-segment crystallites, respectively; △*H*_m,s_ and △*H*_m,h_: melting enthalpy of the soft- and hard-segment crystallites, respectively.

**2.3 NMR, IR and HR-MS spectra**

**Figure S38.** ^1^H NMR spectrum of compound **S2** in CDCl_3_ (400 MHz).

**Figure S39.** ^13^C NMR spectrum of compound **S2** in CDCl_3_ (100 MHz).

**i**

**-OH**

**Figure S40.** ^1^H NMR spectrum of compound **DASA-2** in ACN-*d*_3_ (600 MHz).

**Figure S41.** ^13^C NMR spectrum of compound **DASA-2** in ACN-*d*_3_ (150 MHz).

**Figure S42.** HR-ESI-MS spectrum of compound **DASA-2**.

**Figure S43.** ^1^H NMR spectrum of **DASA-2-HMDI-PCL_1:3_** in tetrahydrofuran-*d*_8_ (400 MHz).

The DASA-2 content in DASA-2-HMDI-PCL_1:3_ was estimated by comparing the integrals of the characteristic aromatic protons of DASA-2 (H_c_, 4H) and the HMDI-derived urethane N-H protons (H_b_, 2H). Based on the theoretical HMDI/DASA-2 molar ratio of 1:0.019, the calculated H_c_/H_b_ integral ratio was 0.038, in close agreement with the experimental value of 0.040.

**3. References**

1.	Guo, Q., Huang, Z., Feng, D., et al., "Precisely regulation of photochromism via absorption-induced photoreaction attenuation and light intensity dependence for time-resolved asymmetric encryption," *Chem. Eng. J.* 499(2024): 156196, <https://doi.org/10.1016/j.cej.2024.156196>.

2.	Clerc, M., Stricker, F., Ulrich, S., et al., "Promoting the Furan Ring-Opening Reaction to Access New Donor-Acceptor Stenhouse Adducts with Hexafluoroisopropanol," *Angew. Chem. Int. Ed.* 60, no. 18 (2021): 10219−10227, <https://doi.org/10.1002/anie.202100115>.

3.	Lui, B. F., Tierce, N. T., Tong, F., et al., "Unusual concentration dependence of the photoisomerization reaction in donor-acceptor Stenhouse adducts," *Photoch. Photobio. Sci.* 18, no. 6 (2019): 1587−1595, <https://doi.org/10.1039/c9pp00130a>.

4.	Sroda, M. M., Lee, J., Kwon, Y., et al., "Role of Material Composition in Photothermal Actuation of DASA-Based Polymers," *ACS Appl. Polym. Mater.* 4, no. 1 (2022): 141, <https://doi.org/10.1021/acsapm.1c01108>.

5.	Fang, L., Lin, Z., Zhang, Y., et al., "Robust, Ultrafast and Reversible Photoswitching in Bulk Polymers Enabled by Octupolar Molecule Design," *Angew. Chem. Int. Ed.* 63, no. 16 (2024): e202402349, <https://doi.org/10.1002/anie.202402349>.

6.	Chen, Q., Diaz, Y. J., Hawker, M. C., et al., "Stable Activated Furan and Donor-Acceptor Stenhouse Adduct Polymer Conjugates as Chemical and Thermal Sensors," *Macromolecules* 52, no. 11 (2019): 4370−4375, <https://doi.org/10.1021/acs.macromol.9b00533>.

7.	Yap, J. E., Mallo, N., Thomas, D. S., Beves, J. E., Stenzel, M. H., "Comparing photoswitching of acrylate or methacrylate polymers conjugated with donor-acceptor Stenhouse adducts," *Polym. Chem.* 10, no. 47 (2019): 6515, <https://doi.org/10.1039/c9py01345h>.
